# Phagocytosis by retinal pigment epithelium and microglia does not affect vision restoration by P3HT nanoparticles in *Retinitis pigmentosa*

**DOI:** 10.1038/s41419-026-08510-w

**Published:** 2026-03-03

**Authors:** Giulia Mantero, Simona Francia, Filippo Galluzzi, Nikita Telitsyn, Dmytro Shmal, Sara Cupini, Edoardo Porzano, Alberto Perna, Matteo Vincenzi, Joao Filipe Ribeiro, Luca Berdondini, Guglielmo Lanzani, Grazia Pertile, Stefano Di Marco, Fabio Benfenati, Elisabetta Colombo

**Affiliations:** 1 Center for Synaptic Neuroscience and Technology, Istituto Italiano di Tecnologia, Genova, Italy; 2 IRCCS Azienda Ospedaliera Metropolitana, Genova, Italy; 3 Department of Experimental Medicine, University of Genova, Genova, Italy; 4 Microtechnology for Neuroelectronics, Istituto Italiano di Tecnologia, Genova, Italy; 5 Center for Nano Science and Technology, Istituto Italiano di Tecnologia, Milano, Italy; 6 Department of Ophthalmology, IRCCS Sacrocuore Don Calabria Hospital, Negrar, Verona Italy; 7 Present Address: IRCCS Azienda Ospedaliera Metropolitana, Genova, Italy

**Keywords:** Neurodegeneration, Macular degeneration

## Abstract

Photoreceptor degeneration in Retinitis pigmentosa (RP) is the most prevalent cause of inherited legal blindness, for which effective visual restoration treatments are still missing. Injectable prosthetic strategies represent a promising tool for vision restoration. We demonstrated that injectable poly(3-hexylthiophene) nanoparticles (P3HT-NPs) promote a sustained visual restoration in Royal College of Surgeons rats, an RP model harboring a mutation that impairs the phagocytic activity of the retinal pigment epithelium (RPE) and microglia, leading to progressive and combined rod/cone degeneration. However, it is unclear whether the efficacy of P3HT-NPs in this model is enhanced by the impairment of RPE and microglial phagocytosis, and thus whether this prosthetic intervention will also be effective in more typical forms of RP that primarily affect rods. Here, we evaluated the efficacy of P3HT-NPs in the pigmented retinal degeneration 10 (rd10) mouse, which carries a recessive missense mutation in the rod phosphodiesterase-6B gene, while retaining a morphologically and functionally intact RPE. We demonstrate that, in this mouse model of RP, P3HT-NPs restore visually driven responses at both subcortical and cortical levels at the end stage of photoreceptor degeneration. Although partial phagocytosis of P3HT-NPs by the RPE occurs, the P3HT-NPs remaining in the outer retina were sufficient to mediate a significant recovery of visual function characterized by complex light-dependent reactivation of the primary visual cortex and formation of implicit visual memories. These results demonstrate that healthy RPE and microglial activities do not compromise the efficacy of the injectable nanotherapeutic strategy, underscoring the clinical potential of P3HT-NPs for visual restoration in late-stage retinal degeneration, which closely mimics the conditions of RP patients undergoing prosthetic interventions.

## Introduction

Sight is one of the most vital senses in humans, enabling us to perceive our surroundings, navigate the world safely, and connect emotionally through visual cues. Among the most prominent and common afflictions that cause visual impairment are inherited retinopathies, such as *Retinitis pigmentosa* (RP; [1–3]), which result in photoreceptor loss and have a worldwide prevalence of over 5 million patients. RP is genetically heterogeneous, as it can be caused by a variety of mutations in many different genes with autosomal dominant, autosomal recessive, or X-linked inheritance. Over 80 genes expressed by photoreceptors or the retinal pigment epithelium (RPE) have been implicated in the generation of RP [3–5]. In its most typical form, degeneration primarily affects rods, causing night blindness and loss of peripheral vision, and eventually cones, leading to complete bilateral blindness.

The field of visual restoration for RP has advanced significantly over the past decade [6, 7]. Several groups have demonstrated a plethora of approaches aimed at providing patients with basic functional vision, thereby restoring some autonomy in their everyday chores and tasks, with each technological step representing a significant stride in quality of life. Gene therapy is by far the disease-modifying therapeutic strategy of choice, as effectively demonstrated by the approval of *voretigene neparvovec* (Luxturna) for biallelic RPE65 mutations. Although other ongoing clinical trials are targeting other mutated genes, limitations apply due to the large number of mutant genes that can cause RP and the necessity of very early intervention before degeneration has irreversibly progressed [8, 9]. Additionally, the highly polygenic etiology of RP makes precision-targeted gene therapy economically unviable for producers and/or patients due to unfavorable economies of scale [4]. Against these odds, in recent years, other genetic approaches based on the transduction of second or third-order retinal neurons with microbial opsins to make them directly light-sensitive yielded promising results in experimental models of RP [10–13]. However, the first-in-man application of optogenetics did not meet expectations, requiring light intensification and achieving only partial rescue of contrast sensitivity and spatial resolution [14]. Cell therapy with stem cells, photoreceptor precursors, or RPE cells is also emerging as a potentially promising intervention for vision restoration [15, 16]. Although these strategies have yielded promising results in animal models of RP, clinical trials are still in their early stages, and risks associated with incomplete differentiation cannot be completely ruled out (for review, see [17]).

When photoreceptor degeneration is complete, the most effective strategy is the implantation of a retinal prosthesis that translates light into an electrical stimulation of inner retinal neurons spared by degeneration [18]. While some neuroprosthetic strategies in this field interface directly with the visual cortex, also applying to other forms of blindness that hit retinal ganglion cells (RGCs) [19, 20], most focus on replacing the function of lost retinal photoreceptors, either by directly stimulating RGCs and optic nerve fibers in case of epiretinal multielectrode chips, or by stimulating second-order retinal neurons to reactivate the processing activities of the neuroretina in case of subretinal photovoltaic devices [21, 22]. Retinal prostheses can be made of either inorganic or organic photosensitive materials. Among inorganic prostheses, the epiretinal *Argus II* and the subretinal devices *Alpha AMS* and *Prima* have shown the most promise [23–25]. In addition, semiconductive conjugated polymers used in organic solar cells have been shown to be effective as a subretinal planar prosthesis in recovering light sensitivity in preclinical models of RP [26, 27]. Apart from the advantages in biocompatibility, flexibility, and the absence of a power supply that characterize organic devices [28], all planar prosthetic approaches suffer from limited spatial resolution and a restricted visual field.

Nanotechnology offers considerable promise for the treatment of nervous system diseases [29–31]. The key advantage of nanotechnology applied to retinal prosthetics is that the nanodevices, scaled down to dimensions smaller than those of single neurons, can achieve single-cell spatial resolution, an essential requirement for effective visual restoration in RP. Indeed, a new frontier in nanotechnology applied to visual restoration was represented by the application of semiconductive conjugated polymers in the form of subretinally injectable nanoparticles (NPs) as light-induced charge generators to activate second-order retinal neurons [17, 30, 32]. We have reported that injectable colloidal suspensions of NPs comprised of conjugated poly(3-hexylthiophene) (P3HT) in either pristine [33–35] or composite architectures [36, 37] are effective in rescuing the visual function of albino Royal College of Surgeons (RCS) rats [38, 39] in both early and late stages of degeneration. In this model of RP, P3HT-NPs did not promote retinal inflammation, remained confined to the external retina, making synaptic-like contacts with bipolar and horizontal cells (BCs and HCs, respectively; [35]). P3HT-NPs were not cleared out or degraded over time by blood circulation, remaining in the subretinal space with unaltered amounts and activity up to 8 months after surgical injections in the RCS rat [33].

These very promising results, however, leave open questions related to their translatability to the cure of RP, irrespective of the underlying mutation. In fact, RCS rats carry a biallelic *loss-of-function* mutation of the *Mertk* gene encoding Mer tyrosine kinase (MerTK), a member of the TAM receptor family (that also includes Tyro3, Axl). MerTK plays a crucial role in regulating phagocytosis in a variety of phagocytic cells, including the RPE and microglial cells in the retina, where it is highly expressed. In the RPE, MerTK serves as a fundamental receptor for the phagocytosis of spent photoreceptor outer segments by RPE short microvilli, also known as sheaths [40]. Phagocytosis is triggered by the binding of RPE ανβ5 integrin to phosphatidylserine exposed on the outer segment membrane and the activation of the RPE focal adhesion kinase. This kinase phosphorylates the phagocytosis receptor MerTK, which, by stimulating actin polymerization and recruiting effectors, produces the engulfment and internalization of shed discs [40]. As photoreceptor survival critically depends on the turnover of their used external discs to prevent accumulation of phototoxic damage due to rhodopsin photobleaching [40, 41], the integrity of RPE is essential to prevent photoreceptor degeneration. Disruption of this housekeeping process congests the outer segment layer microenvironment with unprocessed cellular debris, resulting in the combined degeneration of rods and cones. MerTK is also a key regulator of microglial activity, particularly in triggering non-inflammatory, homeostatic phagocytosis for clearing various debris [42, 43]. Thus, the beneficial effects of the injected P3HT-NPs in the RCS rat, and their persistence in the subretinal space, could, in principle, be attributable to the absence of phagocytic activity by the dysfunctional RPE and microglial cells in this rat strain. While several *MERTK* mutations have been identified in human RP [44], in most cases, the RP-causing mutated gene is exclusively expressed in rods, triggering primary rod degeneration followed by delayed secondary degeneration of neighboring cones [1, 4].

Here, to validate the translational potential of P3HT-NPs, we investigated their fate and efficacy in the presence of healthy RPE and microglia. To this end, we assessed the extent of visual restoration in the homozygous retinal degeneration-10 (rd10) mouse, characterized by a mutation in the *Pde6b* gene that encodes the rod catalytic subunit of cGMP phosphodiesterase-6B. This mouse model recapitulates the most frequent form of human RP with precocious rod degeneration that eventually involves cones, in the presence of an intact RPE [45, 46]. We demonstrate that P3HT-NPs spread out to cover most of the subretinal space after a single subretinal injection in 10- to 14-month-old rd10 mice that reached an end-stage of the disease. While only a minority of P3HT-NPs were phagocytosed by the RPE and microglia, the portion remaining in the outer retina led to a restoration of light sensitivity, visual acuity, and visually driven behavior to levels comparable to age-matched wild-type (WT) animals. The recovery of visual performance was associated with a complex light-dependent reactivation of the primary visual cortex layers and formation of implicit visual memories. Our findings demonstrate that healthy RPE and microglia do not compromise the efficacy of the injectable nanotherapeutic strategy, offering new insights into the clinical potential of P3HT-NPs for restoring vision in late-stage human RP.

## Materials and methods

### Production and characterization of polymeric NPs

P3HT was synthesized by oxidative polymerization of 3-hexyl-thiophene (3HT; 1 g in 40 mL of CHCl_3_) with ferric chloride (Alfa Aesar, Haverhill, MA), according to a procedure [47] that displayed high reproducibility in the characteristics of the polymer (such as regio-regularity, as estimated from ^1^H-NMR, dispersity, and spectroscopic features). P3HT-NPs were obtained from freshly prepared P3HT solution in common organic solvents using the reprecipitation technique in the absence of surfactants, as previously described [48, 49]. Briefly, P3HT (8 mg) dissolved in 300 μL tetrahydrofuran (Sigma-Aldrich, Milano, Italy) was added dropwise via a Hamilton syringe to 4 mL of sterilized MilliQ water under magnetic stirring. The obtained suspension was subjected to dialysis (12,000-dalton cutoff) against 2L of sterile MilliQ water for two days to remove the residual organic solvent. The entire process was conducted under sterile conditions. As a non-photosensitive control, silicon dioxide NPs (SiO_2_-NPs; R-L3235, Microparticles GmbH, Berlin, Germany) of comparable size (nominal diameter: 200 nm) were used. These NPs were provided in a 5% aqueous suspension (w/v) and subjected to autoclave sterilization. They exhibit high monodispersity, with a coefficient of variation of 2.3%, a spherical morphology, excellent optical transparency, and mechanical and thermal stability. The surface of SiO_2_-NPs is functionalized with Si-OH groups, which facilitates the formation of numerous hydrogen bonds that contribute to their hydrophilic behavior. The size and concentration of P3HT-NPs in distilled water were subjected to NP tracking analysis (NTA) using the NanoSight NS300 (Malvern Panalytical Ltd., Malvern, UK), resulting in a hydrodynamic diameter of ~180 nm and a concentration range between ~10^10^ NPs/mL corresponding to ~1 mg/mL P3HT. For transmission electron microscopy (TEM) imaging, samples were prepared by dropping 5 µl of P3HT-NP suspension on 150-mesh copper grids coated with ultrathin holey carbon film (Sigma-Aldrich, Milano, Italy), and imaged with a 1011 TEM (JEOL Ltd., Tokyo, Japan) (Fig. S1).

### Ethical approval and animal handling

All animal manipulations and procedures were conducted in accordance with the guidelines established by the European Community Council (Directive 2014/26/EU of 4 March 2014) and were approved by the Italian Ministry of Health (Authorization # 355/2023-PR). Dystrophic rd10^–/–^ and healthy rd10^+/+^ WT mice in the C57BL6/J background were provided by The Jackson Laboratory (Bar Harbor, ME) and Charles River Laboratories (Calco, Italy). All animals were bred under standard conditions, with *ad libitum* access to food and water, in a 12 h/12 h light/dark cycle. Experimental groups were randomly selected, with a balance of females and males aged 10-14 months, and the experimenter was blinded to group allocation.

### Subretinal surgery

Rd10 mice of either sex aged 10–14 months were anesthetized with an intramuscular administration of xylazine (5 mg/kg) and ketamine (50 mg/kg). Tropicamide eye drops (1% v/v) were administered just before the beginning of the surgery to dilate the pupil. First, a drop of ophthalmic gel was applied to the eye, and a round glass (5 mm in diameter) was placed on top of it. This prevented the eye from drying, allowing us to visualize the retina through the lens. Using a 30-gauge sterile needle, a hole was punctured at the scleral-corneal limbus. The injection was then performed using a 34-gauge blunt-pointed needle connected to a 10-μL Hamilton syringe as follows: the needle was inserted through the hole in the limbus, crossing the vitreous space, taking care to avoid the lens. The needle was used to puncture the retina and access the subretinal space gently. A volume of 1 μL of NP suspension (either SiO_2_ or P3HT, 1 mg/mL) was gradually injected using an automatic pump at a rate of 200 nL/s to avoid a traumatic retinal detachment. Finally, the needle was slowly withdrawn, allowing the retinal puncture to self-seal [50]. Antibiotic and corticosteroid eye drops (tobramycin and dexamethasone) were applied as postoperative prophylaxis.

### Optomotor response test

Measurement of the Optomotor Response (OMR; PhenoSys GmbH, Berlin, Germany) was performed in rd10 mice at 30-60 DPI (Fig. S2). The system automatically detects and measures reflexive head movements in response to moving patterned visual stimuli. The stimulus is presented on four screens mounted around an elevated circular platform on which the animal is placed. Two mirrors are attached to the floor and the lid to create an optical illusion of infinite depth, discouraging the animal from jumping off the platform. A camera automatically tracks body/head movements in response to shifting pattern gratings (black/white bars) of different spatial frequencies. The stimulation protocol included the spatial frequencies 0.012, 0.05, 0.1, 0.15, 0.2, 0.3, and 0.4 c/deg. Each spatial frequency was randomly presented for 60 s at a speed of 12° per sec, and data were collected at least four times for each spatial frequency. This information is then used by the software to determine visual performance automatically (qOMR scores; [51]). The OMR score represents the ratio of concordance to discordance in body/head movements with respect to the moving patterns displayed on the system screens surrounding the animal [52]. The score of 1.0 was considered the threshold for visual perception of a given spatial frequency.

### Light-cued classical conditioning

The classical conditioning (CC) test apparatus (Med Associates Inc., Fairfax, VT) comprises an environmental chamber equipped with a grid designed for administering a mild foot shock. Test sessions were recorded using a camera mounted on the front door and subsequently analyzed through integrated software to identify and quantify behavioral freezing responses. rd10 mice were tested at 60-90 DPI (Fig. S2). The protocol consisted of three distinct phases:

#### Conditioning

The conditioning session began with a 2-min habituation period, allowing animals to explore the environment freely. Then, a sequence of five 2-s white light flickering periods (100 ms, 22 lux, @ 5 Hz, 50% duty cycle) separated by 2-s dark intervals (total: 18 s) was presented as the conditioned stimulus (CS). A mild foot shock (0.75 mA) was administered during the final light stimulus of each sequence as the unconditioned stimulus (US). Each mouse underwent seven CS-US pairings separated by intervals of varying duration [53]. To assess the US-CS associative learning, freezing behavior during each US-CS pairing was quantified.

#### Cue test

Three hours later, the Cue test session was conducted using the same apparatus. To prevent context-US association and selectively evaluate the CS-US association, the chamber was modified by covering the grid floor with a smooth white plastic sheet and replacing the arena with black and white striped walls. Additionally, a new aromatic odor (vanilla, Sigma-Aldrich) and two curved walls were introduced. After 5-min habituation to the modified chamber, the test began with 2 min in the dark without stimulation, followed by 3 min of CS (100 ms flash, 22 lux, @ 5 Hz for 2 s, 50% duty cycle). Freezing behavior was quantified during the 2-min dark and 3-min CS periods to assess the animal’s perception of the light stimulus.

#### Context test

The context session, using the same environment as the conditioning session, was carried out the day after the cue test. Each subject was placed in the chamber for 5 min without CS and US, during which freezing behavior was recorded.

Before each test session, animals underwent 1 h of dark adaptation, with all experiments conducted in darkness. The freezing behavior for each test session was expressed as the percentage of freezing time relative to the total session time.

### Recordings of visually evoked potentials

At 90-120 DPI (Fig. S2), rd10 mice were anesthetized with isoflurane (2.5% induction, 1.5% maintenance in oxygen). Anesthesia remained stable during the experiment, and body temperature (36–37 °C) was monitored. The animal was fixed in a stereotaxic apparatus, and a hole in the skull was drilled in correspondence with the binocular portion of V1 (Oc1b). After exposure of the brain surface, the dura mater was gently removed. A glass micropipette (2–4 MΩ) filled with 3 M NaCl was inserted into Oc1b (between 2.5–3 mm medio-lateral from the intersection of the sagittal and lambdoid sutures). Both eyes were maintained wet with saline solution (NaCl 0.9%), fixed, and opened with adaptable metal rings.

#### Flash visually evoked potentials (fVEPs)

Sensitivity to light was evaluated by recording fVEPs in Oc1b at a depth of about 400 µm in response to full-field flashes of white light (wavelength 400–700 nm; [26, 33, 54, 55]). Visual stimuli (100 ms, 1 Hz) were generated at a luminance of 120 cd/m^2^ by a ViSaGe MKII Stimulus Generator (Cambridge Research Systems Ltd., Cambridge, UK), connected to a monitor (20 × 22” area, 100% brightness and contrast) at a 25-cm distance.

#### Patterned visually evoked potentials (pVEPs)

Spatial acuity was evaluated by measuring pVEPs in response to horizontal sinusoidal contrast-reversing gratings (“patterns”) of increasing spatial frequencies at 1 Hz (from 0.015 to 1 c/deg of visual angle). Signals were amplified and band-pass filtered (0.1–100 Hz) using a NeuroLog system (Digitimer Ltd., Hertfordshire, UK) and then digitized through a multifunction I/O device from National Instruments (Austin, TX). Signal acquisition was performed using MATLAB R2019b software (MathWorks Inc.). pVEPs were analyzed on averaged traces of 200 sweeps, in which peak detection (peak-to-baseline amplitude) was set above 2-fold the standard deviation of the noise, and latency was measured from stimulus onset to peak time using OriginPro2020 (v2020; SR1, 9.7.0.188; OriginLab Corporation). The decrease in pVEP amplitude as a function of the spatial frequency was fitted by linear regression, and the data were plotted on a semi-logarithmic scale. Visual acuity was then estimated by extrapolation to the X-axis intercept. At the end of each session, control recordings were performed by covering the eyes with black tape to rule out electrical artifacts.

### In vivo multielectrode recording of the V1 cortex

At 90-120 DPI (Fig. S2), rd10 mice were anesthetized, prepared, and maintained for surgery as described above. The 256-channel Simultaneous Neural Active Pixel Sensor (SiNAPS) CMOS probe, shown in Fig. S6 (Corticale s.r.l., Genova, Italy; [56]), was inserted to a depth of 3 mm at a location 0.4 mm rostral and 2.7 mm lateral to lambda. Recovery of visual perception was assessed by recording cortical activity in primary visual areas (0–1 mm depth) in response to full-field white light flashes (100 ms, 1 Hz, wavelength 400–700 nm, 120 cd/m^2^).

For VEP analysis, signals were low-pass filtered at 300 Hz and notch-filtered at 50 Hz to eliminate power line noise. VEPs were detected on average traces (50 trials) from channels within the primary visual cortex (V1, 350/550-µm depth) using peak detection analysis; peak-to-baseline amplitudes were assessed in a 75/150-ms window post-stimulus onset, with a detection threshold set at >2 standard deviations above baseline. Spectral analysis was performed on low-pass filtered signals using the MATLAB function *pspectrum*, and Z-scores ((f(t)-F_0_)/std(F_0_)) were calculated for the temporal evolution (5-ms bins) of the power spectral density within the peristimulus time window (–0.1 to 0.4 s relative to flash onset), using the pre-stimulus interval as baseline. For light-evoked multi-unit response analysis, data from the SiNAPS probes were band-pass filtered (300 Hz-5 kHz) using custom MATLAB scripts and spike-sorted with Kilosort 3. Neuronal units with a mean firing rate <0.2 Hz were excluded. Peristimulus time histograms (PSTHs) were calculated by averaging single-unit spike responses across 50 trials, with a bin width of 10 ms. Z-scores were calculated from firing rate smoothed traces (50 ms window) by subtracting the mean baseline firing rate from the peri-stimulus firing rate trace and dividing by the baseline standard deviation. Quantification of light-induced spiking activity was performed by calculating the maximum Z-score in the post-stimulus window (150-500 ms).

### Retina immunohistochemistry

#### Retina dissection

After the electrophysiological recordings (Fig. S2), animals were euthanized by CO_2_ inhalation followed by cervical dislocation. Eyes were enucleated, with attention paid to maintaining the eye orientation during processing. Eyes were fixed overnight in 4% paraformaldehyde in 0.1 M phosphate-buffered saline (PBS). The following days, eyes were cryoprotected by equilibration with 15%, then 30% sucrose solution in PBS after being extensively washed in 0.1 M PBS. Eyecups were obtained by removing the cornea, the iris, and the lens.

#### Whole-mount and transversal retina sections

The eyecup was incised with a scalpel blade, cutting it into 4-5 petals to flatten the retina. The whole mount was incubated in free-floating for 30 min with bisbenzimide (1:300) for nuclear labeling and then rinsed three times with 0.1 M PBS. The whole mount was placed between two coverslips (60 × 40 mm), and the P3HT-NP distribution was assessed by confocal microscopy of the intrinsic fluorescence of P3HT. For transversal retina sections, the eyecup was embedded in OCT freezing medium, frozen in dry ice, and sectioned at 18 μm using a cryostat.

#### Immunofluorescence and confocal fluorescence microscopy

Sections were collected on gelatin- and polylysine-coated (Sigma-Aldrich) glass slides and stored at −20 °C until further processing. For morphological analyses, sections were washed 3 times in 0.1 M PBS to remove any trace of freezing medium. For all the morphological analyses, sections were first incubated with 10% normal goat serum (NGS) at room temperature (RT) for 1 h to block non-specific antibody binding. Then, they were incubated overnight at 4 °C with the primary antibody of interest, at the concentrations reported in Table 1. To remove excess primary antibodies, samples were rinsed 3 times in 0.1 M PBS and then incubated for 1 h at room temperature with species-specific secondary antibodies together with bisbenzimide nuclear labeling (1:300; Hoechst 33258; Sigma-Aldrich), rinsed 3 times in 0.1 M PBS, and mounted with Mowiol. The following secondary antibodies were used: Alexa Fluor 488 (1:500, A11029, Invitrogen), Alexa Fluor 568 (1:500, A11075, Invitrogen), and Alexa Fluor 647 (1:500, A21245, Invitrogen).

**Table 1:** Primary antibodies used for retina immunohistochemistry.

Primary antibodies	Supplier	Cat. No.	Host	Dilution
Anti-Cone Arrestin	Merck	AB15282	Rabbit	1:500
Anti-Rhodopsin	Abcam	ab221664	Rabbit	1:500
Anti-GFAP	SySy	173011	Guinea Pig	1:1000
Anti-Iba-1	Wako	01919741	Rabbit	1:250
Anti-CD68	Abcam	AB53444	Rat	1:500
Anti-Parvalbumin	SySy	195004	Guinea Pig	1:250
Anti-PKCα	Santa Cruz	8393	Mouse	1:250
Anti-Tyr Hydroxylase	Merck	AB152	Rabbit	1:250
Anti-NeuN	SySy	266004	Guinea Pig	1:200

### Image analysis

Retinal sections were imaged using a Leica SP8 confocal microscope (Leica Microsystems). All morphometric analyses of the retina were performed by imaging and averaging of 230 × 230 × 18 μm central and peripheral Z-stacks with an XY resolution of 1024 × 1024 pixels and a Z-step of 700 nm, encompassing slices that passed through both the primary injection site and the optic disc. Acquisition parameters were kept constant throughout the imaging sessions for comparison purposes.

#### Analysis of the NP distribution

P3HT-NPs imaging was done on Hoechst 33258-stained whole-mount preparations and transversal sections of the mouse retinas by acquiring the intrinsic P3HT fluorescence (λ_EX_, 514 nm; λ_EM_, 650–750 nm) with the SP8 confocal microscope. P3HT-NP cluster diameters and nearest neighbor distances (NND) were extracted from binarized (thresholding method: *Moments*) 290 × 290 × 50-μm Z-stack volumes of retinal sections, acquired at 1024 × 1024 resolution. Cluster major diameters were grouped into 250 nm bins and converted into frequency distributions by normalizing to the total number of clusters. NPs coverage was assessed from whole-mount retinas, with areas measured using a manually defined region of interest (ROI). NP extension boundaries were determined on binarized Z-max projections of the NP fluorescence using the *boundary* function in MATLAB (MathWorks Inc., Natick, MA).

#### Extent of retinal degeneration and rewiring

Rods in dystrophic groups were individually counted, and the density in non-dystrophic groups was measured by averaging cell counts from 3 ROIs in the ONL. This density was then used to estimate a total count per field based on the total ONL area. Cones were manually counted for all experimental groups. The rewiring of rod BC axons, identified as PKCα-positive cells, was quantified by measuring the angle at which their axons intersected a line perpendicular to the retinal layering. Further analysis of the IPL, where BC axons terminate, included assessing the width profile of the terminal layer and calculating the standard deviation of this profile as a metric of layer disruption. Morphometric changes in parvalbumin-expressing amacrine cells type II and RGCs were evaluated by counting positive cells in the INL and ganglion cell (GCL), respectively [57, 58]. To assess preservation of the retinal output, in addition to the subpopulation of PV-positive RGCs, NeuN-positive cells were quantified at the level of the GCL. For dopaminergic amacrine cells, tyrosine hydroxylase (TH) expression was assessed by measuring integrated density across all retinal layers. The maximum intensity projection (Z-max) of the TH signal was used for segmentation (thresholding method: *Moments*) to measure the area occupied by dopaminergic amacrine cells and for quantifying the integrated density.

#### Analysis of NP phagocytosis by RPE and microglia and proinflammatory activity

RPE cells were identified by Texas Red-Phalloidin (T7471; 1:40 dilution from a 200 U/mL solution in DMSO; ThermoFisher Scientific, Waltham, MA) labeling of F-actin, which highlighted the actin-dense apical microvilli. P3HT-NP clusters within RPE cells were identified from binarized images and spatially categorized as described above. The percentage of P3HT-NP internalization within the RPE was calculated as the ratio of fluorescent NP clusters found inside the RPE to the total number of clusters detected. Microglial cells, identified by Iba-1 immunoreactivity, were counted across all retinal layers. To assess their state of activation, we evaluated the extent of ramified or ameboid shape by using the Sholl analysis and the circularity index. The evaluation of the degree of process ramification using Sholl analysis was performed with the SNT plugin in the ImageJ Fiji distribution. Iba-1-positive cells were segmented using a custom ImageJ pipeline that involved binarization (utilizing the *Moments* method), manual curation, skeletonization, and dilation. The minor diameter of the cell soma was used as the starting radius, and intersections were counted at increasing distances from the soma using concentric circles with 1 µm diameter increments. The resulting intersection profile, plotted as a function of distance from the soma, was fitted with a log-normal curve. The maximal branching span was determined at the point where the fitted curve reached 50% of its peak value (half-width). The circularity index was calculated using the Circularity plugin in the ImageJ Fiji distribution [circularity = 4π(area/perimeter^2^)]. A circularity value of 1.0 indicates a perfect circle. As the value approaches zero, it means an increasingly elongated polygon. Microglial phagocytic activation in response to NP injection was assessed by double immunolabeling with Iba-1 and CD68 antibodies, followed by quantification of double-positive cells. The extent of NP internalization was assessed by measuring the colocalization between P3HT fluorescence and Iba-1-positive profiles, using the Manders’ coefficient calculated with the JaCoP plugin in ImageJ Fiji. Images were manually thresholded before analysis, using the *Moments* auto-thresholding method as a starting point. The integrated density of GFAP immunoreactivity labeling Müller cells/astrocytes was obtained by averaging measurements from 6 ROIs in both the IPL and ONL on a slice sum projection of each field.

#### Analysis of RPE cell morphology

The integrity of RPE cells was analyzed in phalloidin-stained whole-mount retina preparations using ImageJ to perform manual segmentation. Segmented images were subsequently analyzed using a Fiji (https://imagej.net/Fiji) script that detects cells and computes a set of geometrical parameters (area, perimeter, major/minor ellipsis axes, circularity index). Finally, the number of neighbors for each cell was calculated using the BioVoxxel_toolbox plugin that generated the corresponding heatmaps.

### Statistical analysis

The sample size required for the in vivo experiments (n) was predetermined using the G*Power software for an ANOVA test with four experimental groups, considering an effect size of 0.40-0.60, an alpha (type I error) of 0.05, and a 1-β (type II error) of 0.90, based on similar experiments and preliminary data. Experimental data are expressed as means ± sem throughout, with n being the number of independent animals. Normal distribution was assessed using D’Agostino-Pearson’s normality test. Two-tailed tests have been employed for all statistical evaluations. To compare two normally distributed sample groups, either a paired or an unpaired Student’s *t*-test was used. The Mann-Whitney *U*-test was used to compare two sample groups that were not normally distributed. To compare more than two normally distributed sample groups, one-way or two-way ANOVA was followed by Tukey’s, Holm-Sidak’s or Fisher’s LSD multiple comparison test. To compare more than two non-normally distributed sample groups, the non-parametric Kruskal-Wallis test followed by Dunn’s multiple comparison test was used. The discrete Kolmogorov-Smirnov test was used to compare the frequency distribution of a given visual response between two experimental groups. Statistical analysis was carried out using OriginPro (OriginLab Corporation), MATLAB R2019b (MathWorks Inc.), and GraphPad Prism 10 (v10.2.3; GraphPad Dotmatics, Boston, MA).

## Results

### P3HT-NPs do not affect photoreceptor degeneration or cause inflammation in the rewired retina of rd10 mice at end-stage RP

In rd10 mice, rod degeneration starts as early as the 14th postnatal day and is complete at 2 months of age, while cones undergo a delayed secondary degeneration with precocious loss of external segments, followed by the progressive degeneration of the cell bodies in the outer nuclear layer (ONL) that is almost complete around 10-12 months of age [45, 46, 59, 60]. To reliably test visual restoration in fully degenerated retinas that completely lost light sensitivity, in the absence of confounding effects due to residual photoreceptors, either inert size-matched glass NPs (SiO_2_) or photoactive P3HT-NPs of 180/200-nm diameter (P3HT; Fig. S1) were transvitreally injected in the subretinal space of 10/14-month-old dystrophic rd10 mice, which were followed for up to 120 days post-injection (DPI) with behavioral and electrophysiological tests (Fig. S2).

**Fig. 1:**
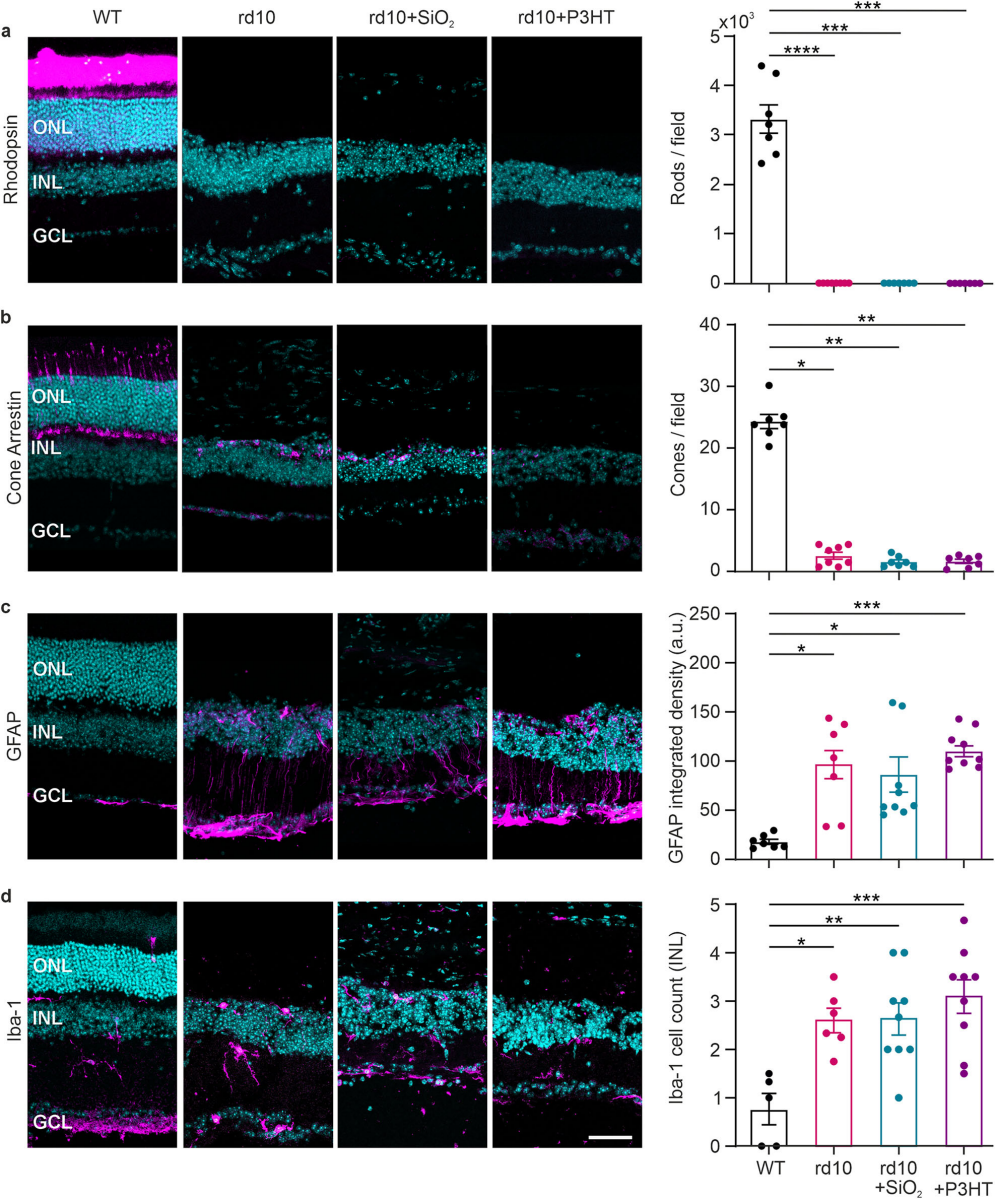
Injected P3HT-NPs do not affect photoreceptor degeneration and are not pro-inflammatory in old rd10 mice. Transverse retinal sections taken at 120 DPI from 14/18-month-old healthy controls (WT), untreated dystrophic rd10 mice (rd10), and dystrophic rd10 mice injected with either SiO_2_ (rd10 + SiO_2_) or P3HT (rd10 + P3HT) NPs (see Fig. S1). Retinal sections were immunolabeled with antibodies to rhodopsin for rods (**a**), cone arrestin for cones (**b**) GFAP for Müller cells/astrocytes (**c**) and Iba-1 for microglia (**d**). In all representative images shown on the left, the specific immunostaining is shown in magenta, while Hoechst 33258 nuclear staining is shown in cyan. Scale bars, 100 µm. **a**, **b** *Photoreceptors*. At this advanced stage of degeneration, dystrophic retinas were almost completely devoid of photoreceptors under all experimental conditions. Bar plots on the right show the respective cell counts. Very rare arrestin-positive cone cell bodies can be observed in the total absence of external segments. *n* = 7, 8, 7, and 7 mice for WT, rd10, rd10 + SiO_2,_ and rd10 + P3HT groups, respectively. **c** *Müller cells/astrocytes*. The bar plot on the right shows the integrated density of GFAP immunofluorescence in the ONL. *n* = 7, 7, 9, and 9 mice for WT, rd10, rd10 + SiO_2,_ and rd10 + P3HT groups, respectively. **d** *Microglia*. The bar plot on the right reports the counts of Iba-1 immunoreactive microglial cells in the ONL. Dystrophic retinas exhibit an inflammatory state due to degeneration, regardless of the treatment. *n* = 5, 6, 9, and 9 mice for WT, rd10, rd10 + SiO_2,_ and rd10 + P3HT groups, respectively. All data represented as bar plots are means ± sem with superimposed individual data points. ONL, outer nuclear layer; INL, inner nuclear layer; GCL, ganglion cell layer. *p < 0.05, **p < 0.01, ***p < 0.001, ****p < 0.0001; one-way ANOVA/Holm-Sidak’s or Kruskal-Wallis/Dunn’s tests.

To assess the degree of photoreceptor degeneration in this end-stage RP and exclude the possibility that surgical intervention or electrical stimulation in degenerating retinas has a protective/trophic effect on photoreceptors [61, 62], we labeled rods and cones with antibodies to rhodopsin and cone arrestin, respectively (Fig. 1). In dystrophic rd10 mice injected at 10-14 months of age and analyzed 4 months later, rods were totally absent (Fig. 1a), while very rare cone cell bodies were still present, embedded in a thinned inner nuclear (INL), but totally devoid of external segments (Fig. 1b). No differences were observed in the photoreceptor populations between the dystrophic groups, irrespective of whether they were non-injected (rd10), injected with inert NPs (rd10 + SiO_2_), or injected with photoactive P3HT-NPs (rd10 + P3HT), ruling out any trophic effect of the injection surgery or of P3HT (Fig. 1a, b).

Since, at advanced degeneration stages, the inner retina is subjected to a complex remodeling secondary to the prolonged denervation that can potentially interfere with the visual restoration process [63–66], we verified the extent of neuroretina rewiring. In age-matched WT and homozygous rd10 mice (14–18 months of age; Fig. S3a), we analyzed the number of NeuN-positive cells (mostly RGCs), parvalbumin (PV)-positive neurons in the GCL and INL layers, the tyrosine hydroxylase (TH)-positive amacrine cells, and the protein kinase Cα (PKCα)-positive rod BCs by immunohistochemistry. While NeuN- and PV-positive neurons (mostly RGCs with few AII-amacrine cells in the mouse; [57, 58]) were not affected by degeneration, a significant decrease in TH-positive dopaminergic amacrine cells was observed (Fig. S3b–g). Notably, rod BCs were seriously disorganized, with a marked deviation of their axon orientation from the right angle, and with a thinner and less organized IPL (Fig. S3h, i).

To evaluate whether the injection of NPs had a proinflammatory effect on the retina, we immunolabeled transverse retinal sections for the glial fibrillary acidic protein (GFAP) and the ionized calcium binding adaptor molecule-1 (Iba-1), biomarkers of active Müller cells/astrocytes and microglia, respectively (Fig. 1c, d). The intensity of GFAP immunoreactivity in the outer nuclear layer (ONL) was similarly increased in all rd10 groups because of the ongoing photoreceptor degeneration, irrespective of whether they were non-injected, sham-injected, or injected with P3HT-NPs (Fig. 1c). Similarly, the population of Iba-1-positive microglial cells in the INL was increased in all rd10 groups with respect to the WT control, indicating that the presence of either SiO_2_ or P3HT NPs is devoid of proinflammatory effects (Fig. 1d).

Moreover, Iba-1-positive microglial cells were subjected to the morphometric analysis of their pro-inflammatory propensity (Fig. S4a). When activated, microglial cells lose their quiescent, ramified morphology to assume a pro-inflammatory, amoeboid-like shape. We tested whether the presence of SiO_2_ or P3HT NPs in the outer retina had any effect on the transition of microglia between these two states. Sholl analysis, which evaluates the extent of ramifications arising from the cell body, showed that microglial cells were highly ramified in WT retinas, while the presence of degeneration per se, independent of the injection of NPs, significantly decreased ramifications, indicating a degeneration-induced inflammatory state (Fig. S4b). We also analyzed the morphology of the Iba-1-positive cells, using the circularity index as a shape descriptor, ranging from 1 (full circularity) to 0 (fully asymmetric shape). The shape of microglial cells confirmed the results of the Sholl analysis, showing highly asymmetric shapes in WT animals and a shift to ameboid shapes in dystrophic animals, regardless of whether they were non-injected or injected with NPs (Fig. S4c). Taken together, the immunohistochemical data confirm that the subretinal injection of either SiO_2_- or P3HT-NPs in the rd10 mouse produces no trophic or pro-inflammatory effects.

### Injected P3HT-NPs widely distribute in the subretinal space of end-stage rd10 mice

**Fig. 2:**
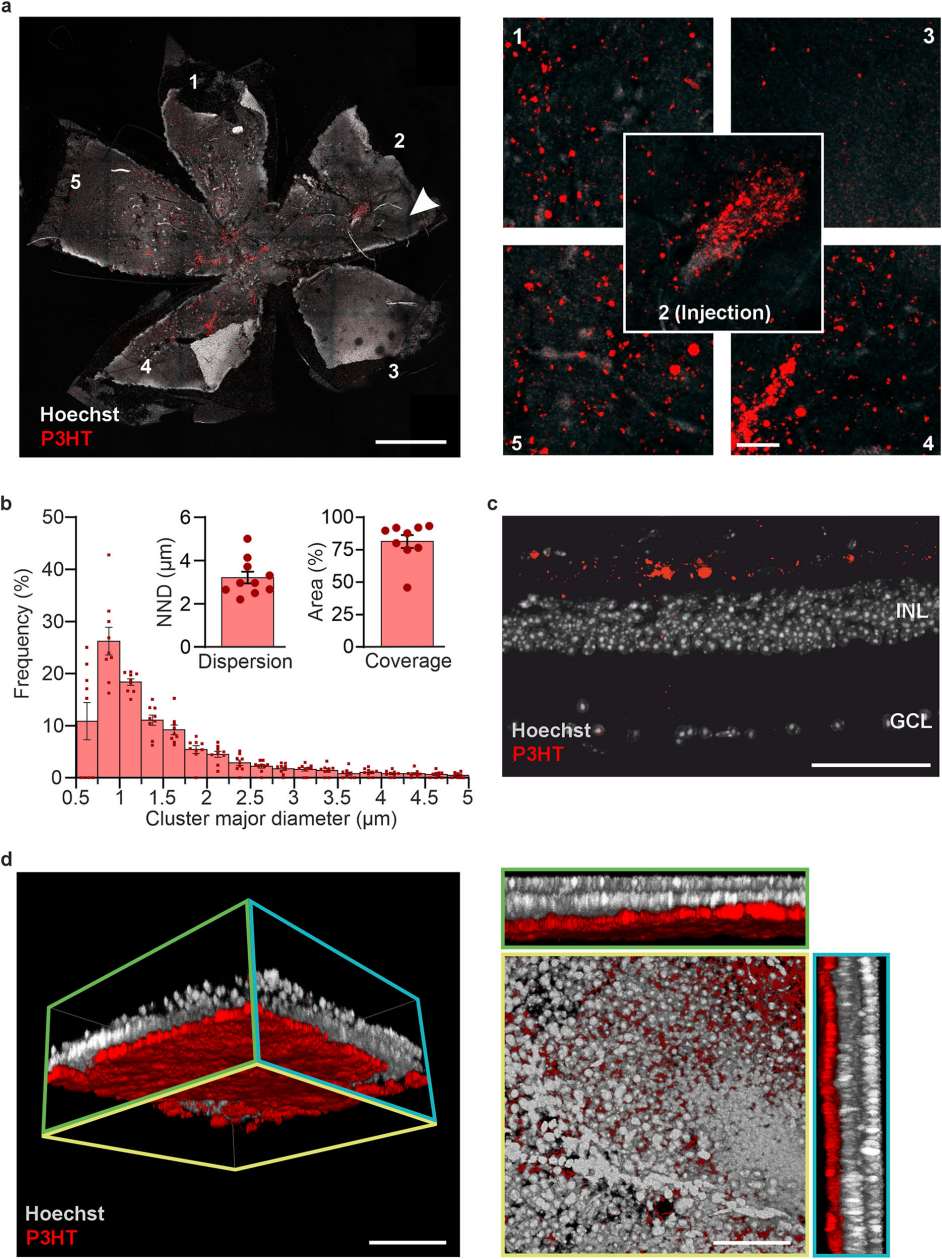
P3HT-NPs evenly distribute and achieve an extensive retinal coverage after a single subretinal injection in the old rd10 mouse. **a** Left: Whole-mount confocal image of a homozygous rd10 mouse retina (nuclei stained with Hoechst 33258 in white) injected with P3HT-NPs (red). Numbers correspond to the areas shown at higher magnification in the right panel. The arrowhead indicates the site of injection. Scale bar, 1 mm. Right: Higher magnification images of whole mount petals corresponding to the numbers in the left panel. Scale bar, 50 µm. **b** Histogram describing the distribution of the major diameter of P3HT-NP clusters in the retina (250 nm bins). Data are means ± sem with overlaid individual data points. Inset: Bar plots of the mean (± sem) nearest neighbor distance (NND, left) and of the mean (± sem) percentage of the whole mount retinal area covered by P3HT-NPs, delimited by the outermost detectable P3HT-NP clusters. **c** Transverse section of an injected rd10 retina, showing the distribution of P3HT-NPs in the outer retina. Nuclei are stained with Hoechst 33258 (white). Scale bar, 100 µm. **d** Left: 3D reconstruction of whole-mount confocal retinal image adjacent to the injection site (cell nuclei in white) showing the subretinal localization of the injected P3HT-NPs (red) obtained by fluorescence confocal microscopy under super-resolution conditions. Scale bar, 100 μm. *Right:* Z-axis magnification of each retinal layer and the orthogonal XZ, YZ projections of the retina injected with P3HT-NPs (red). Scale bar, 100 μm.

To evaluate the retinal coverage and the NP permanence in the retina, we assessed the spread and clustering of P3HT-NPs in the subretinal space 4 months after a single microinjection (120 DPI), leveraging the intrinsic red fluorescence of P3HT. Retinas were explanted and imaged in whole-mount and transverse configurations for the intrinsic P3HT fluorescence using super-resolution confocal microscopy (Leica SP8/HyD with Lightning deconvolution, see Methods). Distribution area analysis of the flat mounts revealed that a single injection spread P3HT-NPs over 80% of the subretinal space, with the highest density concentrated around the injection site (Fig. 2a, b). Analysis of the size of P3HT-NP clusters revealed a main peak at 0.75–1 µm diameter, with >98% of the NP clusters sized below 2 µm. The mean nearest neighbor distance (NND), a measure of the granularity of the NP distribution on the retinal surface, was 3 µm, indicating the potential for activating the inner retina at the single-cell level (Fig. 2b). Morphometric analysis on transverse retinal sections revealed that P3HT-NPs remained strictly confined to the outer retinal layers, namely the outer plexiform layer (OPL) and the INL, with no tendency to migrate towards the innermost retina layers (Fig. 2c). This was particularly evident in the 3D super-resolution reconstruction of a retina patch, as well as in the transverse XZ and YZ planes (Fig. 2d).

### Functional RPE and microglial cells phagocytose only a small fraction of P3HT-NPs in end-stage rd10 mice

Contrary to the *Mertk* mutation that underlies the RCS rat model, the *Pde6b* biallelic missense mutation of the rd10 mouse does not affect RPE function and microglial phagocytosis. This makes it an ideal model to investigate the fate of subretinal nanotherapeutics in the presence of *Mertk*-expressing phagocytic RPE and microglia, which is the case in the majority of the human RP variants [40, 42, 43].

**Fig. 3:**
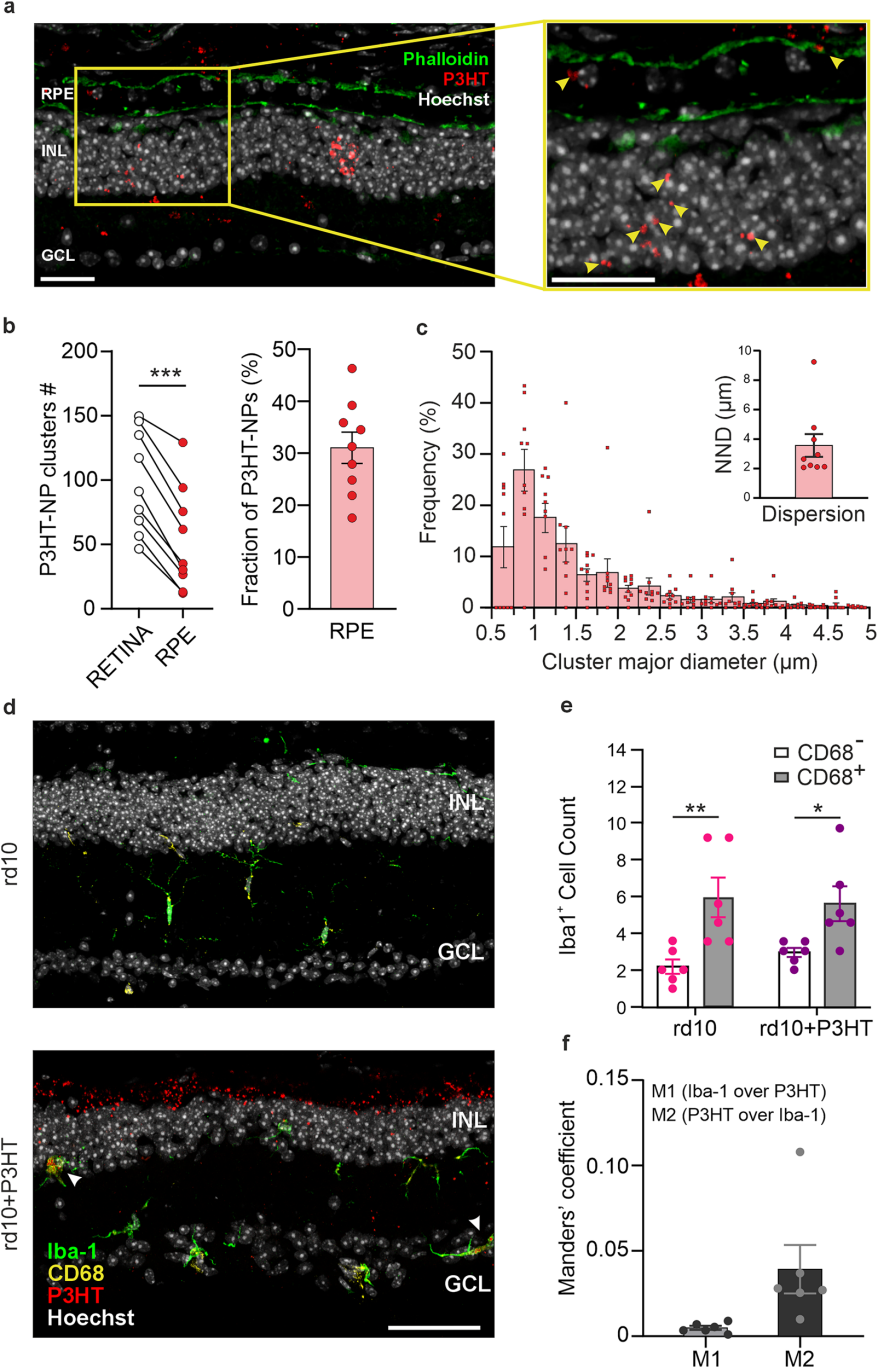
P3HT-NPs are partially captured by the RPE and not phagocytosed by microglial cells in old rd10 mice. Retinal sections were taken from non-injected (rd10) and P3HT-NP-injected (rd10 + P3HT) dystrophic mice at 120 DPI (14/18 months of age). **a** *Left:* Representative confocal image (z-projection) from an rd10 + P3HT mouse retina labeled with Hoechst 33258 for cell nuclei (white), Texas Red-Phalloidin for RPE visualization (green*)*, and P3HT-NP intrinsic fluorescence (red). *Right:* higher magnification of the retinal area delimited by the yellow square. RPE, retinal pigment epithelium; INL, inner nuclear layer; GCL, ganglion cell layer. Scale bar, 25 µm. **b** Quantification of P3HT-NP clusters in the RPE. *Left:* Comparison of the number of P3HT-NP clusters inside the RPE and in the retina. *Right:* Mean ± sem percentage of P3HT-NP clusters (with overlaid individual data points) internalized by the RPE with respect to the total P3HT-NPs injected in the retina (*n* = 9 mice). **c** Histogram describing the distribution of the major diameter of P3HT-NP clusters within the RPE and their corresponding nearest neighbor distance (NND; *inset*). Data are means ± sem with overlaid individual data points (*n* = 9 mice). The size distribution of P3HT-NP clusters and their NND closely match the corresponding values measured in the retina (see Fig. 1). **d** Representative immunostaining of a non-injected rd10 retina (*top;* rd10) and a P3HT-NP-injected rd10 retina (*bottom*; rd10 + P3HT) labeled with Hoechst 33258 for cell nuclei (white), Iba-1 for microglia (green), and CD68 for microglial phagocytic activation (yellow). In the injected retina, P3HT-NPs are also visible (red). Arrowheads indicate double-stained Iba-1/CD68 microglial cells with internalized NPs. Scale bar, 50 µm. **e** Quantification of the number of CD68-positive (gray bars) and CD68-negative (open bars) Iba-1-stained microglial cells across all retinal layers. In both dystrophic retinas, a significant increase in CD68-positive cells is observed due to degeneration. However, no differences were observed in the number of CD68-positive cells in the absence or presence of P3HT-NPs (*n* = 6 for both rd10 and rd10 + P3HT groups). **f** Quantification of the P3HT/Iba-1 fluorescence. Bar plots (means ± sem with individual data points) show the Manders’ M1 coefficient (expressing the proportion of the total Iba-1 immunoreactive area colocalizing with the P3HT-positive area) and M2 coefficient (expressing the proportion of the total P3HT fluorescent area colocalizing with the Iba-1 immunoreactive area) for both markers. (*n* = 6 rd10 + P3HT mice). ns not significant; *p < 0.05, **p < 0.01, ***p < 0.001; paired Student’s *t* test (**b**); two-way ANOVA/Tukey’s *t*ests (**e**).

To better visualize the interactions between P3HT-NPs and the RPE, we performed confocal imaging of transverse retinal sections stained with fluorescent phalloidin to label the actin-rich dense microvilli that characterize RPE cells and the intrinsically fluorescent P3HT-NPs (Fig. 3a). We found that ~30% of the injected P3HT-NPs, evaluated based on the number of fluorescent NP clusters, were taken up by the RPE, while the remaining ~70% persisted in the outer retina (Fig. 3b), free to form hybrid contacts with the tight-knit, surviving second-order neurons. The size of P3HT-NP clusters within RPE cells and their NND were superimposable with the same parameters determined in the outer retina (main peak at 0.75–1 µm diameter; NND ∼ 3.5 µm; Fig. 3c; compare with Fig. 2b). To establish whether the internalized P3HT-NPs were harmful to the RPE, the morphology of RPE cells was evaluated from phalloidin-stained whole-mount retinas of WT, non-injected rd10 mice, and rd10 mice injected with P3HT-NPs. For each RPE flat mount image, cell borders were recognized and segmented, and the number of neighboring cells, cell density, and circularity index were computed (Fig. S5a–c). We found that the WT RPE cells had a higher number of neighboring cells, a higher cell density, and a higher circularity index (approaching a hexagonal shape) compared with rd10 mice, irrespective of whether they were untreated or injected with P3HT-NPs (Fig. S5d–f). This indicates that the phagocytized NPs do not have, per se, adverse effects on RPE cells. No significant changes were also observed in the cell shape (circularity index) across the three experimental groups.

Although phagocytosis of P3HT-NPs by Iba1-positive cells was not observed in RCS retinas [33, 35, 37], the lack of MerTK also hinders the phagocytic activity of microglia that physiologically express high MerTK levels [42, 43]. Thus, we also checked whether the P3HT-NPs remaining extracellular were captured by microglial cells. As phagocytically active microglia overexpress the lysosomal/endosomal glycoprotein CD68, which is involved in phagocytic activity, we double-stained microglial cells in the outer retina for Iba-1 and CD68 in untreated and P3HT-NP-injected rd10 retinas (Fig. 3d). In both experimental groups, there was a comparable increase in the number of Iba-1-positive cells that also expressed CD68, indicating the presence of a phagocytic activation toward the cell debris resulting from degeneration. However, the presence of subretinal P3HT-NPs was not associated with an increased CD68 expression by microglia, indicating that no phagocytic activation is induced by the presence of NPs in the outer retina (Fig. 3e). To directly assess the presence of P3HT-NPs inside microglial cells, we used super-resolution confocal microscopy and Manders’ coefficient analysis to quantify the extent of overlap between Iba-1 immunoreactivity and P3HT fluorescence. We found that < 1% of the P3HT fluorescence overlapped with Iba-1 immunoreactivity (M1 coefficient) and only ∼4% of Iba-1 immunoreactivity was occupied by P3HT fluorescence (M2 coefficient), indicating a negligible phagocytosis of P3HT-NPs by microglial cells, notwithstanding their degeneration-induced activation (Fig. 3f).

### P3HT-NPs improve spatial resolution-driven behavior in end-stage rd10 mice

**Fig. 4:**
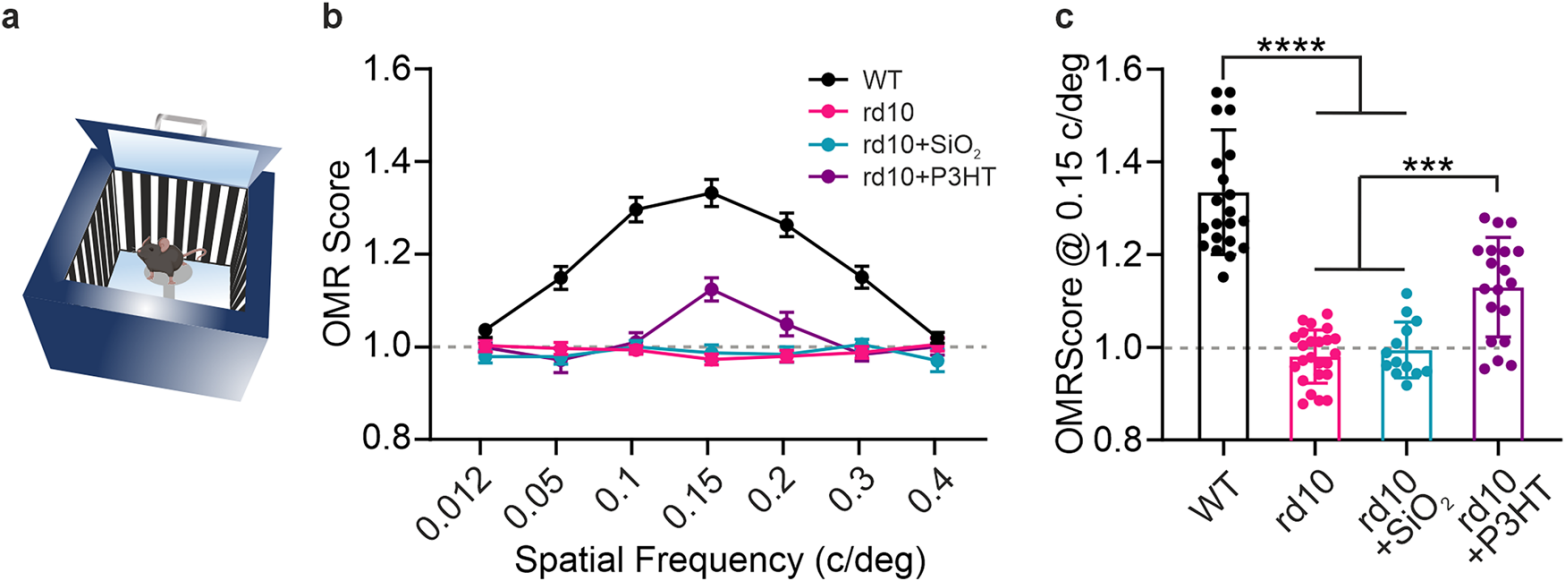
P3HT-NPs improve spatial discrimination of old rd10 mice in the optomotor response test. **a** Schematic representation of the apparatus for the optomotor response (OMR; for details, see Materials and Methods). **b** OMR scores (means ± sem) across all tested spatial frequencies for the 4 experimental groups: WT mice, untreated rd10 mice (rd10), and rd10 mice sham-injected (rd10 + SiO_2_) or injected with P3HT-NPs (rd10 + P3HT). The rd10 + P3HT group showed a significantly increased score at 0.15 c/deg compared to rd10 and rd10 + SiO_2_ mice. The dashed line (OMR score = 1) represents the visual threshold. **c** Bar plots of the individual OMR scores (means ± sem with superimposed individual experimental points) for the 4 experimental groups at 0.15 c/deg. The rd10 + P3HT group showed a marked improvement in visually driven behavior compared to either the rd10 or the rd10 + SiO_2_ group. **p < 0.01, ****p < 0.0001; one-way ANOVA/Tukey’s tests (*n* = 22, 24, 13, and 19 for WT, rd10, rd10 + SiO_2_, and rd10 + P3HT, respectively).

To investigate the behavioral correlates of the spatial/pattern perception, WT and rd10 mice that were non-injected, sham-injected, or injected with P3HT-NPs were subjected to the optomotor response (OMR) test at 30-50 DPI (Fig. S2). The OMR is a physiological reflex induced by moving gratings of decreasing spatial frequency, which, if resolved, elicits a chasing movement of the head and neck aimed at keeping the image stable on the retina. Driven initially by the neuronal input from direction-selective RGCs, this reflex analyzes subtle changes in the spatial resolution and performance of retinal neurons (Fig. 4a; [67]). Our analysis, performed in 11- to 15-month-old mice, revealed that WT mice exhibited significant OMR responses across a wide range of spatial frequencies (from 0.05 to 0.3 c/deg), which were significantly above the threshold OMR score of 1, representing indifference towards the stimulus. On the contrary, both non-injected (rd10) and sham-injected (rd10 + SiO_2_) rd10 mice displayed no significant OMR responses over the full range of spatial frequencies, with OMR scores that did not deviate from the threshold line. Notably, rd10 mice injected with P3HT-NPs (rd10 + P3HT) demonstrated a significant increase in OMR score at 0.15 c/deg (Fig. 4b). The statistical analysis of the visual performance of the four experimental groups at 0.15 c/deg revealed a highly significant OMR recovery in rd10 + P3HT mice compared to either rd10 or rd10 + SiO_2_ mice, which approached, but remained significantly lower than, the OMR response of WT mice (Fig. 4c).

### P3HT-NPs restore light-induced implicit memory in end-stage rd10 mice

**Fig. 5:**
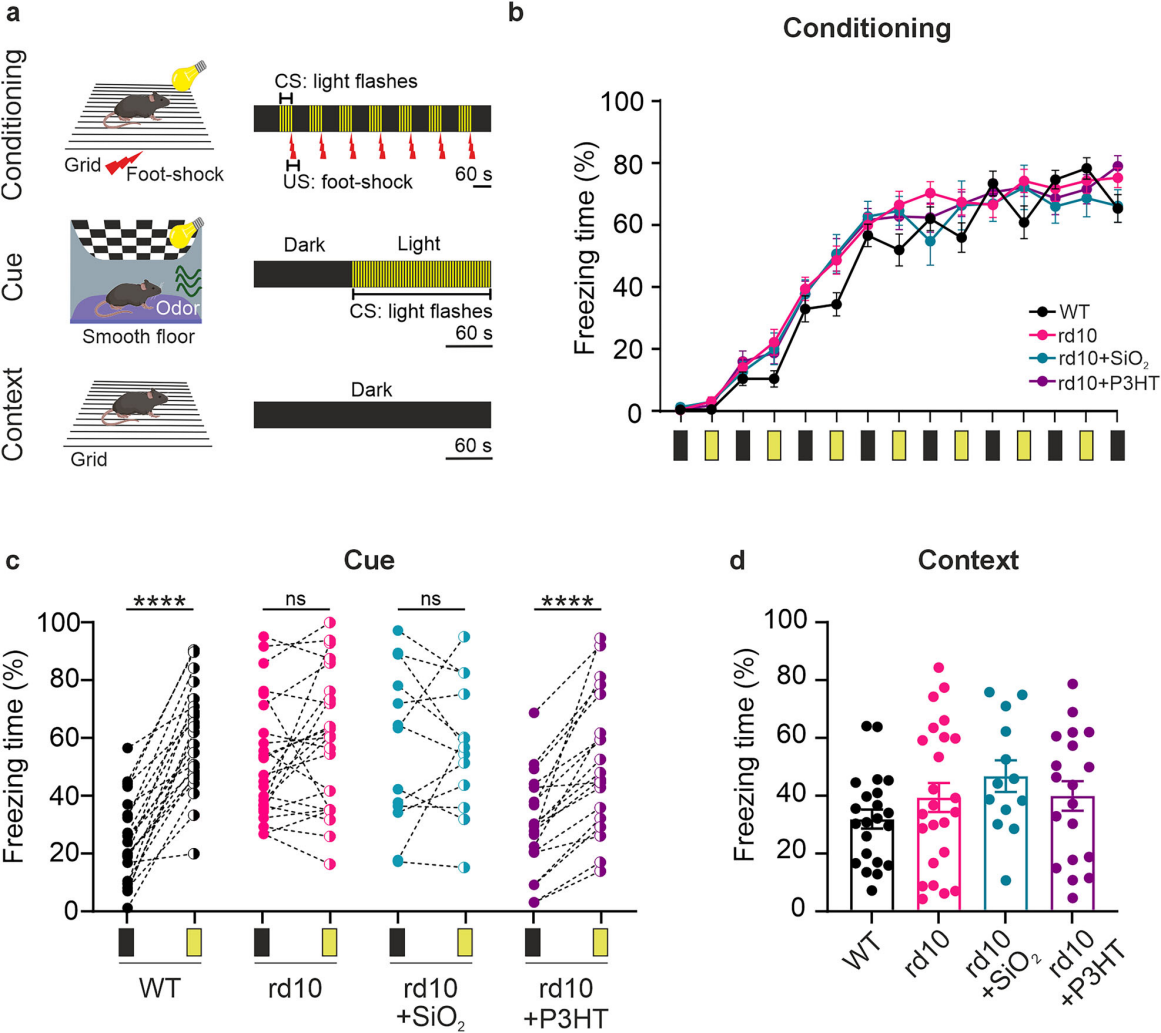
P3HT-NPs improve the visual performance of old rd10 mice in a classical conditioning behavioral test. **a** Schematic representation of conditioning cue and context protocols (for details, see Materials and Methods). *Top:* During the conditioning session, mice are placed in the dark chamber of the apparatus for 2-min habituation and then subjected for 9 min to 7 sequences of white light flashes as the conditioned stimulus (CS; 100 ms, 22 lux, @ 5 Hz for 2s) paired with the unconditioned one (US; mild foot shock, 0.75 mA) administered during the final CS of each sequence. *Middle:* During the cue test session, mice underwent 5-min habituation in the dark to explore a novel context, after which CS was presented for 3 min. *Bottom:* During the context session, mice were placed in the same context as the conditioning session, but without the presentation of the CS. **b** Progressive increase of the conditioned fear reaction to the light stimulus at 30 DPI in sighted mice (WT), untreated homozygous rd10 mice (rd10), and homozygous rd10 mice that were subretinally injected with either inert SiO_2_-NPs (rd10 + SiO_2_) or photoactive P3HT-NPs (rd10 + P3HT). The conditioned fear response was evaluated as the percentage freezing time recorded during each CS-US pairing. All experimental groups learned the conditioned response similarly. Black rectangles indicate dark time intervals; yellow rectangles indicate light stimulation time intervals. Data are means ± sem. **c** During the cue phase, WT and rd10 + P3HT demonstrated a higher tendency towards freezing behavior when the CS was presented. In contrast, no difference in freezing behavior was measured between light CS and dark in rd10 or rd10 + SiO_2_ mice. **d** In the context phase, no difference in the freezing behavior was observed between the experimental groups. Data are means ± sem with superimposed individual experimental points. Statistics: (**b**) ns not significant, two-way ANOVA; (**c**) ns, ****p < 0.0001, paired Student’s *t* tests; (**d**) ns one-way ANOVA (*n* = 22, 24, 13, and 19 for WT, rd10, rd10 + SiO_2,_ and rd10 + P3HT, respectively).

To demonstrate the effect of P3HT-NPs on the recovery of cortical processing related to light perception and implicit memory formation in blind mice, we performed light-cued classical conditioning at 60-90 DPI (Fig. S2; [35, 37]). This behavioral test relies on the induction of a freezing behavior due to the expectation of a mild foot shock (unconditioned stimulus, US), which the animal has been trained to temporally associate with a light neutral stimulus (conditioned stimulus, CS) (Fig. 5a; see “Materials and Methods”).

The US-CS association requires neural integration occurring at the level of higher brain centers, involving projections from the lateral geniculate nucleus to V1/V2 cortex, visual association area TE2, perirhinal cortex, and the amygdala [68]. During the conditioning training phase, all experimental groups exhibited a comparable increase in freezing behavior over time, indicating similar aptitude in acquiring the freezing response to foot shock (Fig. 5b). During the cue session, WT mice exhibited a marked increase in freezing time during CS presentation with respect to darkness, whereas both non-injected (rd10) and sham-injected (rd10 + SiO_2_) dystrophic mice showed no freezing responses to CS. Strikingly, dystrophic mice injected with P3HT-NPs (rd10 + P3HT) displayed a behavior that was indistinguishable from that of WT mice, with a marked increase in the freezing response to the light CS in 100% of the injected mice (Fig. 5c). This demonstrates that both WT and rd10 + P3HT mice successfully associate the CS with the US, indicating not only the restoration of light sensitivity to levels comparable with those of WT mice, but also the reactivation of cortical processing of visual information necessary to consolidate implicit visual memories. Finally, the freezing behavior recorded in the context session, used as a negative control, was comparable across all experimental groups, indicating equal hippocampus-dependent association of the US with the context, regardless of visual function (Fig. 5d).

### P3HT-NPs restore visually evoked potentials in end-stage rd10 mice

**Fig. 6:**
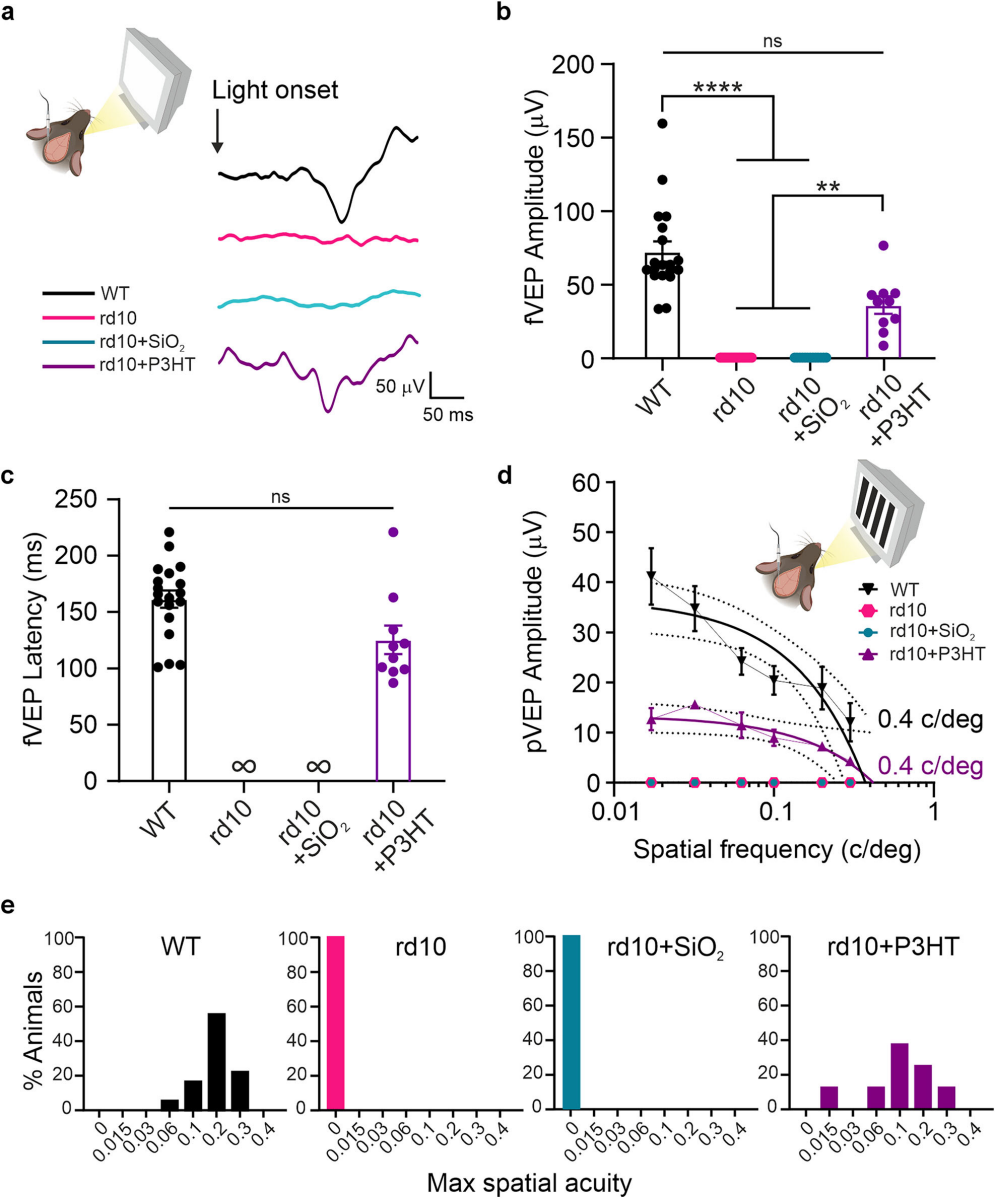
P3HT-NPs improve the visual performance and visual acuity of old rd10 mice. **a** Experimental setup for visually evoked potential (VEP) recordings in the binocular portion of V1 (Oc1b) in response to light stimuli (120 cd/m^2^, 100 ms, 1 Hz) and representative flash VEPs (fVEPs) recorded in WT mice, untreated rd10 mice (rd10), and rd10 mice injected with either SiO_2_-NPs (rd10 + SiO_2_) or P3HT-NPs (rd10 + P3HT) at 90-120 DPI. **b** Bar plots of the fVEP amplitude (means ± sem with superimposed individual experimental points) recorded in Oc1b. VEP responses are totally absent in untreated or sham-injected dystrophic rd10 mice. rd10 + P3HT mice display an improvement of light sensitivity, with VEP amplitudes that are not significantly different from those recorded in age-matched WT mice. ns, not significant; **p < 0.01, ****p < 0.0001, Kruskal-Wallis/Dunn’s tests. **c** Bar plots of the fVEP latency (means ± sem with superimposed individual experimental points) recorded in Oc1b. Latencies observed in age-matched WT mice are comparable to those recorded in rd10 + P3HT mice. The rd10 and rd10 + SiO_2_ mice exhibit an infinite latency, showing no response to the light stimulus. ns, not significant; Mann–Whitney’s *U*-test. **d** Amplitude of VEP responses (means ± sem) to patterned stimuli at increasing spatial frequency (pVEP). The mean visual acuity, estimated as the X-intercept of the individual VEP amplitude decays, reveals a significant recovery in rd10 + P3HT mice that reaches the acuity value of WT mice, albeit with lower amplitude pVEPs. No responses to patterned stimuli of any spatial frequency were recorded in rd10 and rd10 + SiO_2_ mice. **e** Frequency distribution (percent of the total number of mice per group) of the maximum individual spatial acuity displayed by the 4 experimental groups. The frequency profiles of WT and rd10 + P3HT groups are closely similar, indicating a full recovery of visual acuity (*p* = 0.17, discrete Kolmogorov-Smirnov test). Sample size in (**b**, **c**): *n* = 19 and 10, for WT and rd10 + P3HT, respectively. Sample size in (**d**, **e**): *n* = 18 and 9, for WT and rd10 + P3HT, respectively.

To examine the extent of activation of the primary visual cortex (V1) by the recovered visual signals, we performed flash visually evoked potential (fVEP) recordings in the binocular Oc1b visual area in response to light flashes at the age of 14/18 months (90-120 DPI; Fig. 6a). Non-injected (rd10) or sham-injected (rd10 + SiO_2_) rd10 mice showed a total lack of cortical responses to light stimuli, while the rd10 mice that were injected with P3HT-NPs 4 months before (rd10 + P3HT) displayed clear cortical responses to light stimuli (Fig. 6a). The analysis of the fVEP amplitude confirmed that rd10 + P3HT mice produced responses that were not significantly different from age-matched WT mice, although with lower amplitudes (Fig. 6b). Consistent with the recovery in fVEP amplitude, rd10 + P3HT mice displayed VEP latencies within the physiological range that were not markedly different from the latencies of WT mice (Fig. 6c).

Next, we performed cortical VEP recordings evoked by patterned visual stimuli at increasing spatial frequency (pVEP; Fig. 6d, e). Both rd10 and rd10 + SiO_2_ mice exhibited no cortical responses to patterned stimuli, confirming the total loss of visual function. In contrast, rd10 + P3HT mice displayed pVEP responses that, notwithstanding the lower amplitude, reached the same estimated visual acuity cutoff at 0.4 c/deg (Fig. 6d). Analysis of the frequency of the individual maximum spatial discrimination in the four experimental groups showed a closely similar distribution pattern between WT mice and rd10 + P3HT mice (Fig. 6e). Altogether, these results show that the P3HT-NPs resident in the outer retina are entirely capable of generating optoelectronic stimulation and light-dependent cortical activation, generating a visual acuity comparable to that of age-matched WT mice.

### P3HT-NPs electrically reactivate V1 cortical layers in response to light stimuli in end-stage rd10 mice

**Fig. 7:**
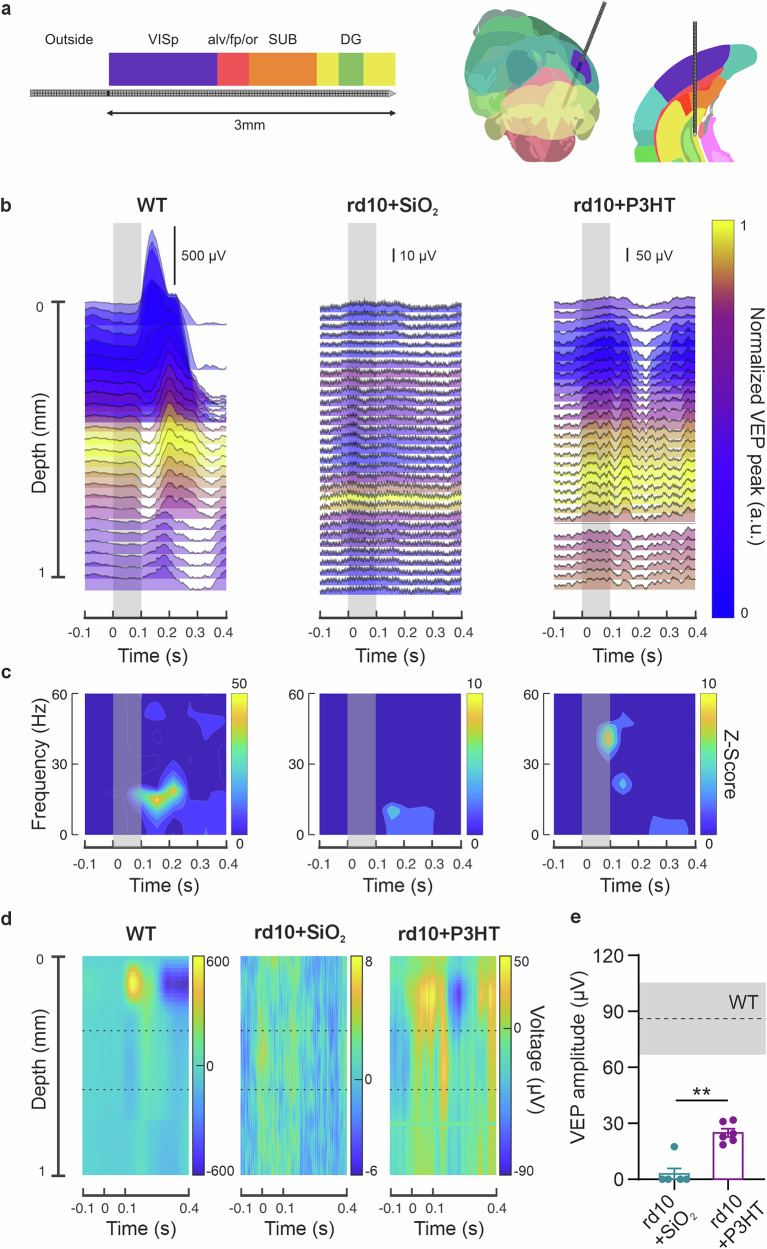
In vivo multielectrode recordings of the rd10 mouse reveal recovery of visual perception in stratified visual areas by P3HT-NPs. **a** Schematic representation of the microelectrode probe used for in vivo acute cortical recordings upon light stimulation. The probe was inserted 3 mm deep at 400 µm rostral and 2700 µm lateral from lambda, thus intersecting several cortical regions: V1 cortex (VISp), *alveus*, *fasciola cinerea*, and optic radiation (alv/fp/or), *subiculum* (SUB), and dentate gyrus (DG). WT and rd10 mice (10–14-month-old) injected with either SiO_2_ (rd10 + SiO_2_) or P3HT (rd10 + P3HT) NPs were implanted with the SiNAPS probe in primary visual areas (0–1 mm depth) and recorded at 120 DPI. **b** Representative low-pass filtered traces of the visually evoked potentials (VEPs) from age-matched WT (*left*), rd10 + SiO_2_ (*middle*), and rd10 + P3HT (*right*) mice. Colors indicate signal voltage in the 75/150-ms window after the light flash (gray shaded box): yellow corresponds to more negative voltage values, while blue corresponds to more positive voltage values within the window of interest. The rd10 + P3HT mouse shows VEP activation at the same spatial level as the WT animal, whereas the rd10 + SiO_2_ mouse does not. **c** Heatmaps of the Z-score temporal frequency profile from a single electrode in layer 4 of the V1 cortex for WT mice (*left*), rd10 + SiO_2_ mice (*middle*), and rd10 + P3HT mice (*right*) during the peristimulus time window (–0.1 to 0.4 s from light flash, gray shaded area). Activation patterns are present in WT and rd10 + P3HT mice but are absent in rd10 + SiO_2_ mice. **d** Heatmaps of the same animals reported in (**b**) and (**c**), showing comparable activation patterns in WT and rd10 + P3HT mice. **e** Quantification of VEP amplitude in layer 4 of the V1 area (350–550 µm depth). The gray shaded area represents the mean ± SEM recorded in WT animals (*n* = 4). Bars indicate the means ± SEM of the VEP amplitude in rd10 + SiO_2_ (*n* = 6) and rd10 + P3HT (*n* = 6) mice. **p = 0.0022; Mann–Whitney *U*-test.

To assess visual perception recovery across the stratified layers of the V1 cortex, we recorded cortical activity in response to light flashes using the high-density cortical probe SiNAPS ([56]; Fig. S6). The 256-channel probe was inserted 3 mm deep, spanning the full depth of the visual cortex and capturing its hierarchical processing architecture (Fig. 7a). In the low-frequency domain, WT mice exhibited robust VEPs, characterized by a clear inversion of the surface-to-depth peak. The largest negative VEP amplitudes were localized to layer 4 (350–550 µm depth), consistent with its role as the main thalamic input layer (Fig. 7b, *left*). Sham-injected rd10 mice (rd10 + SiO_2_) showed no time-locked cortical responses to light stimulation (Fig. 7b, *middle*), while P3HT-NP-injected rd10 mice (rd10 + P3HT) displayed recovery of visual responsiveness. Notably, rd10 + P3HT mice also showed the largest peak VEPs in layer 4, along with preserved sign inversion of the VEP waveform across cortical depth (Fig. 7b, *right)*. In the frequency domain, WT mice exhibited post-stimulus activation in the beta band, associated with attention and cortical engagement. The rd10 + P3HT group also exhibited stimulus-locked frequency responses, although shifted toward the low gamma band, which is more closely linked to sensory processing (Fig. 7c). Given the role of layer 4 in early synaptic processing of visual inputs, we quantified VEP amplitudes in this layer (350–550 µm, delimited by broken lines in Fig. 7d) and found a significant recovery in rd10 + P3HT mice compared to rd10+SiO₂ mice (Fig. 7e). However, the amplitude remained below the range observed in WT mice (gray shaded area).

Since VEPs, as local field potentials, reflect the collective synaptic inputs to a brain region, we also examined the spiking output of individual neurons in response to light flashes (Fig. S7a). In WT mice, light stimulation evoked a robust increase in firing rate. While rd10+SiO₂ mice showed no detectable response, rd10 + P3HT mice exhibited a weaker but consistent increase in firing activity (Fig. S7b, c).

## Discussion

Retinitis pigmentosa is a highly heterogeneous disease, with over 80 genes identified that, if mutated, cause irreversible damage to photoreceptors [3, 5]. Causative genes can be expressed directly by photoreceptors or by the RPE, which is essential for photoreceptor survival. In the advanced stages of RP, once complete photoreceptor degeneration is achieved, the implantation of a retinal prosthesis to trigger light-induced stimulation of inner retinal neurons is a promising therapeutic strategy for recovering visual signals from the retina to the brain. In the rapidly evolving field of prosthetic visual restoration in RP [13], we have recently introduced organic semiconducting NPs [17, 30, 32]. Polymeric NPs can be composed of pristine P3HT, donor-acceptor heterojunctions, or core-shell nanoarchitectures to enhance charge separation activity and thereby increase phototransduction efficiency [36, 37, 49]. Subretinal microinjection of these NPs in the RCS rat model of RP [38], characterized by a rapid rod/cone degeneration, promoted an almost complete restoration of visual functions that was still present in aged RCS rats at the end-stage of RP [33, 35]. The colloidal nature of the “*liquid retina*” preparation offers several advantages over planar prostheses, including broad retina coverage, action at the single-cell level with high potential for spatial resolution, a large surface area to interface with retinal neurons, and a minimally invasive injection procedure [30]. The extent of visual restoration was found to precisely correlate with the number of electrical contacts between NPs and second-order retinal neurons spared by degeneration [35]. Electrostatic and photochemical modelling and electrophysiological experiments demonstrated that the light-dependent pseudo-capacitive charging of conjugated polymer NPs results in depolarization of the neuronal membrane in the presence of a highly resistive cleft [33, 35, 69]. Indeed, owing to the large size (~200 nm), neurons are unable to internalize NPs [70, 71] and instead form tight contacts with their apical surface, characterized by very high resistance with respect to the extra-junctional membrane [33, 34].

One crucial question to address for translating the colloidal strategy to human patients is the fate of NPs and how long they remain in the external retina to exert their activity. The absence of blood vessels in the outer retina helps prevent their clearance by the circulation. However, RPE and microglial cells in the retina act as a tight surveillance of the extracellular environment by actively phagocytosing foreign bodies and cell debris, and therefore represent a potentially effective mechanism for clearing exogenous NPs from the retina. Previous studies in the RCS rat demonstrated that NPs remain in unaltered concentrations and preserve their activity in the outer retina for up to 8 months after a single injection [33]. However, this model, which recapitulates some human forms of RP [39, 44], is not well-suited for studying the long-term NP fate in the external retina, as it is characterized by a primary impairment of RPE phagocytic activity that causes rod/cone degeneration. In fact, the RCS rat is characterized by the biallelic loss-of-function mutation of the *Mertk* gene encoding MerTK, a member of the TAM family tyrosine kinases, that is highly expressed by RPE and microglial cells and is fundamental for the non-inflammatory clearance of various debris, including nanomaterials, by phagocytosis [40, 42, 43]. Thus, given the marked impairment of phagocytosis by RPE and microglia of RCS rats, it was necessary to test the efficacy and fate of P3HT-NPs in an alternative RP model endowed with physiological phagocytic activities of RPE and microglia.

Thus, P3HT-NPs were challenged with the rd10 model of RP, which bears a missense mutation in exon 3 of the rod-expressed gene *Pde6b*, encoding the cGMP phosphodiesterase-6B, that triggers rod degeneration followed by secondary cone degeneration. Mutations in this gene are prevalent in autosomal recessive RP (OMIM180072), and this mouse model effectively recapitulates the most typical forms and clinical course of RP in humans [45, 46, 60]. In rd10 mice, rod degeneration starts at postnatal day (P)14, peaks at P21-25, and is complete at P60. At this stage, only remnants of cones that have lost external segments and exhibit signs of degeneration are present in the outer retina. In this study, to minimize any residual light sensitivity of the retina, we used rd10 mice aged 10–14 months at the time of injection, which were followed up to 14–18 months of age. At this end-stage of the disease, not only has the retina lost any residual light sensitivity, but it has also completely rewired due to the prolonged denervation, losing the physiological synaptic connectivity essential for processing visual signals ([63–66]; our results).

Importantly, RPE and microglia of the rd10 mouse physiologically express MerTK and are thus fully competent for phagocytosis. Indeed, RPE cells were found to internalize P3HT-NPs. However, this activity accounted for only a relatively small fraction (30%) of the injected P3HT-NPs over the significantly long time analyzed (4 months after injection), when compared with an average life expectancy of 18–28 months for rd10 mice. Although RPE cells are affected by the ongoing inflammatory state due to degeneration, their substantially unaltered morphology in untreated and P3HT-NP-injected rd10 mice indicates that the engulfed P3HT-NPs are not harmful to RPE. We also tested whether microglial cells, expressing functional MerTK, were contributing to the clearance of P3HT-NPs from the extracellular space. As expected, the long-standing degeneration had already activated microglial cells, but there was no further increase in number in the presence of P3HT-NPs. More importantly, the presence of P3HT-NPs did not further induce the microglial cells to adopt an ameboid phagocytic shape or increase the expression of the phagocytic activity marker CD68. Moreover, the analysis of the internalized fraction of P3HT-NPs within microglial cells revealed that only <5% was phagocytized. Thus, despite the physiological activity of RPE and microglia, most of the injected P3HT-NPs remained extracellular in the outer retina, engaged in contacting processes and cell bodies of bipolar and horizontal cells, as previously demonstrated [33, 35].

Notwithstanding the partial clearance by RPE and microglia, aged rd10 mice injected with P3HT-NPs yielded a clear-cut restoration of visual performance: (i) they fully recovered the ability to form and recall visual implicit memories generated using a Pavlovian classical conditioning protocol, involving activation of large subcortical and cortical areas [68]; (ii) they displayed optomotor responses to spatial frequencies to which untreated or sham-injected rd10 mice were totally insensitive; (iii) they recovered VEP responses to flash and patterned stimuli and regained a level of visual acuity indistinguishable from age-matched WT controls, in spite of smaller amplitudes of cortical responses, likely due to the extensive retinal rewiring. Additionally, the recovery of near-physiological visual cortex activation in response to light stimuli was thoroughly analyzed using a high-density transcranial probe. This analysis revealed that P3HT-NPs recovered VEP activity, with the largest peak in layer 4, consistent with its role as the main thalamic input layer, and the physiological surface-to-depth peak inversion of the VEP waveforms. While WT mice showed light-evoked activation in the beta band, associated with attention and cortical engagement, the responses of P3HT-NP-injected rd10 mice were shifted toward the low gamma band, more linked to sensory processing [72–74]. The differences in the VEP frequency domains and in the multiunit activity may be attributed to the distinct nature of the bionic signals generated by the NPs, the rewiring of the neuroretina, and the changes in visual cortex connectivity resulting from prolonged signal deprivation. Although very rare residual cone cell bodies entirely devoid of external segments were present, at the age at which NPs were injected (10–14 months), and in the following 4 months of analysis of the visual performance, both untreated and sham-injected rd10 mice were characterized by the total absence of light-cued conditioning, optomotor responses, and VEPs, testifying to the total lack of a residual light-sensitivity or trophic effects of surgery [61, 62]. In summary, the in vivo tests demonstrated that P3HT-NPs resident in the outer retina are entirely capable of generating optoelectronic stimulation that not only recovers light sensitivity of the fully blind retina but also reactivates cortical processing of visual information necessary to consolidate implicit visual memories and achieve spatial discrimination and visual acuity to levels comparable to WT mice.

These results have dual translational significance. Firstly, they extend the validity of the *liquid retina* approach for the light-induced activation of the inner retina to another RP model belonging to a different species and affected by a distinct gene mutation that primarily hits the phototransduction cascade of rods rather than the function of the RPE. Moreover, the progression of RP in the rd10 mouse more closely recapitulates the typical form of human RP, in which degeneration primarily affects rods in the peripheral retina and eventually involves cones, ultimately leading to total blindness. Secondly, we used rd10 mice in their end-stage of RP in which they did not display any residual visual activity: rods were completely absent, very few residual and degenerating cones had lost external segments completely and inner retinal circuits had rearranged and deteriorated, with hardly predictable changes in receptive field organization [63–66]. The persistence of the P3HT-NP effects in these mice indicates that this strategy can be effective in restoring vision, independent of the causative mutation and the stage of the disease.

In conclusion, we demonstrate that organic P3HT-NPs maintain their prolonged effects of restoring vision in RP after a single subretinal administration, in the presence of fully functional phagocytic activity of RPE and microglia, in a different species, in a disease model caused by a distinct mutation directly affecting rod photoreceptors, and in advanced stages of RP with marked rewiring of the neuroretina. Taken together, the data indicated that conjugated polymer NPs represent a clinically effective, highly biocompatible strategy for sustained visual restoration in retinal dystrophies, and that they can efficiently cope with the genetic heterogeneity of RP, with functional or dysfunctional RPE and microglial surveillance, with normal or heavily rewired inner retina.

## Supplementary information

Supplementary Figures

## Data Availability

The experimental data supporting the figures and other findings in this paper are hosted at the Istituto Italiano di Tecnologia and can be accessed by contacting the corresponding authors.
