# Supplementary material for: Phagocytosis by retinal pigment epithelium and microglia does not affect vision restoration by P3HT nanoparticles in Retinitis pigmentosa
Source: Cell Death Dis. 2026 Mar 3;17(1):295. doi: 10.1038/s41419-026-08510-w (PMC13039545; doi:10.1038/s41419-026-08510-w)
Supplement: Supplementary file 1 — Supplementary Figures [file 41419_2026_8510_MOESM1_ESM.pdf]

Phagocytosis by retinal pigment epithelium and microglia does not affect vision restoration by P3HT nanoparticles in *Retinitis pigmentosa*

Giulia Mantero[1]*, Simona Francia[1*#], Filippo Galluzzi[1], Nikita Telitsyn[1,2], Dmytro Shmal[1], Sara Cupini[1], Edoardo Porzano[1,3], Alberto Perna[4], Matteo Vincenzi[4], Joao Filipe Ribeiro[4], Luca Berdondini[4], Guglielmo Lanzani[5], Grazia Pertile[6], Stefano Di Marco[1,3], Fabio Benfenati[1,2*§], Elisabetta Colombo[1,2*]

[1]*Center for Synaptic Neuroscience and Technology, Istituto Italiano di Tecnologia, Genova, Italy*

[2]*IRCCS Ospedale Policlinico San Martino, Genova, Italy*

[3]*Department of Experimental Medicine, University of Genova, Italy*

[4]*Microtechnology for Neuroelectronics, Istituto Italiano di Tecnologia, Genova, Italy*

[5]*Center for Nano Science and Technology, Istituto Italiano di Tecnologia, Milano, Italy*

[6]*Department of Ophthalmology, IRCCS Sacrocuore Don Calabria Hospital, Negrar, Verona, Italy*

* *Equal contribution*

§ *Corresponding author*

\# *Present address:* IRCCS Ospedale Policlinico San Martino, Genova, Italy

SUPPLEMENTARY FIGURES

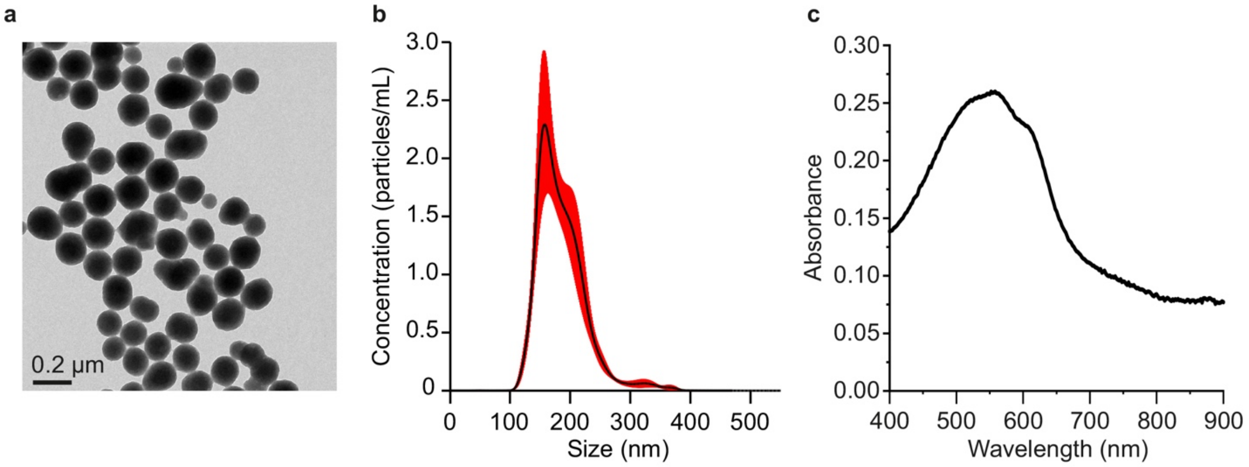

Figure S1. Characterization of the P3HT-NP preparation.
a. TEM micrograph of 5 μl of P3HT-NP suspension on 150-mesh copper grids coated with ultrathin holey carbon film. (Scale bar, 0.2 μm).
b. NTA concentration and size distribution of P3HT-NPs showing an average diameter of ~187 nm.
c. Absorption spectrum of a P3HT-NPs sample in distilled water.

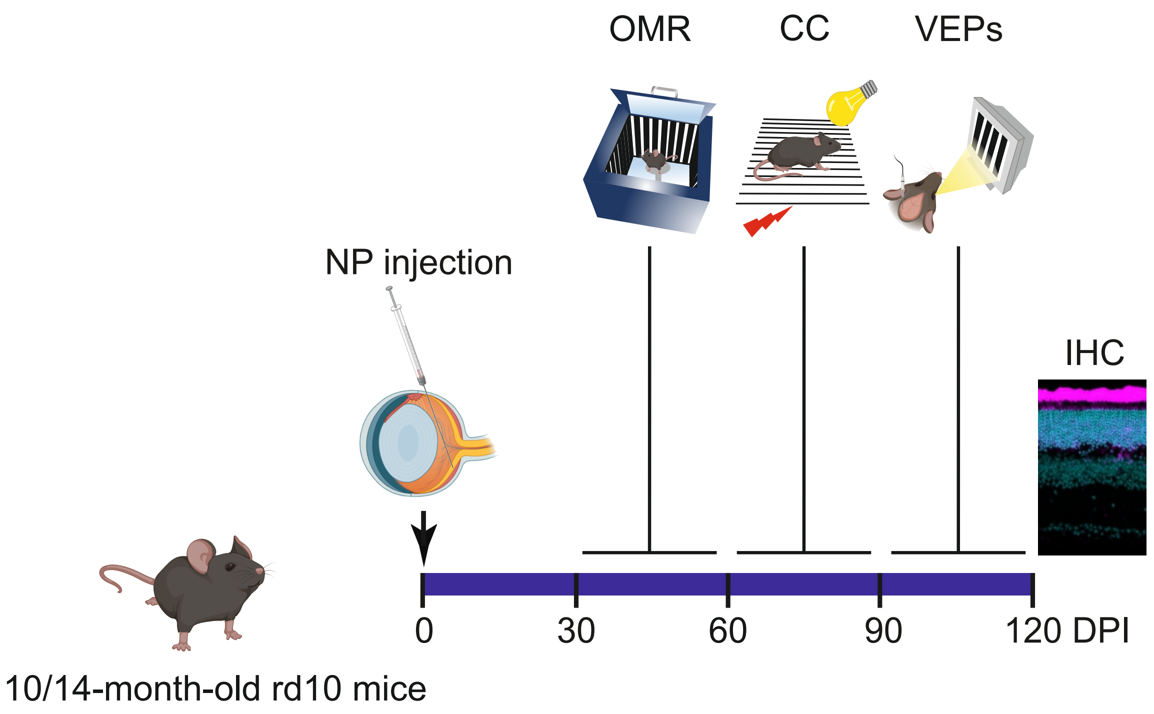

Figure S2. Timeline of experiments for testing visual restoration in aged rd10 mice after the subretinal injection of P3HT-NPs.
Homozygous rd10 mice aged 10-14 months were subretinally injected with either inert SiO_2-NPs (sham) or P3HT-NPs and left to recover from surgery for 30 days. Starting from 30 days post-injection (DPI; 11-15 months of age), behavioral tests for visual perception were conducted: all animals underwent first the optomotor response (OMR) test for spatial resolution perception (30-60 DPI), followed by light-cued classical conditioning (CC) for visually driven fear reactions (60-90 DPI). Between 90 and 120 DPI (up to 14-18 months of age), visually evoked potentials (VEPs) in response to flash and patterned stimuli were recorded, followed by immunohistochemical analysis (IHC) of the explanted retinas.

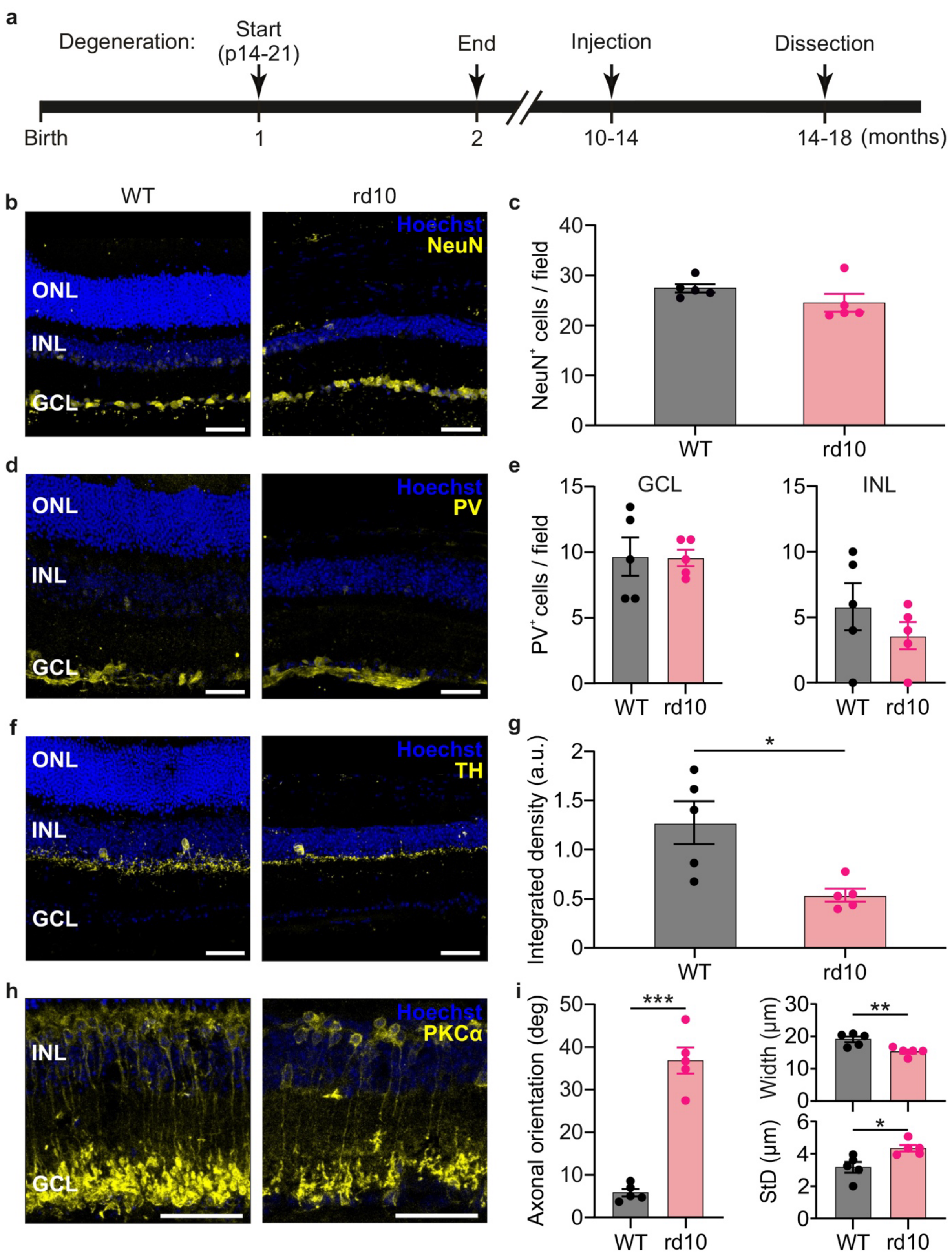

Figure S3. The retina of old rd10 mice has undergone a complex rewiring.

a. In homozygous rd10 mice, rod degeneration starts between postnatal days 14 and 21 (p14-21). By two months of age, the animals are functionally blind: rod degeneration is virtually complete, with only a monolayer of nuclei visible in the ONL. Rare cones devoid of external segments persist for a few months, and the inner retina undergoes a complex rewiring over time (Chang et al., 2002, 2007; Marc et al., 2003; Gargini et al., 2007). To assess the extent of rewiring and the survival of inner retinal neurons up to 14-18 months of age, transverse sections of retinas dissected from dystrophic

(rd10) and age-matched WT mice were processed by immunohistochemistry after the completion of the physiological tests for assessing visual restoration.

b,c. Representative images of NeuN immunoreactivity in age-matched WT and rd10 mice (**b**). The number of NeuN-positive neurons in the GCL was not significantly affected by prolonged degeneration (**c**).

d,e. Representative images of Parvalbumin (PV) immunoreactivity in age-matched WT and rd10 mice (**d**). In the mouse, PV is mainly expressed by a subpopulation of RGCs and, to a lesser extent, by AII amacrine cells (Kim & Jeon, 2006). *Right:* The number of PV-positive neurons in the GCL (RGCs) and INL (AII amacrine cells) was not significantly affected by prolonged degeneration (**e**).

f,g. Representative images of tyrosine hydroxylase (TH) immunoreactivity labeling a subpopulation of dopaminergic amacrine cells in age-matched WT and rdf10 mice (**f**). The integrated density of TH immunoreactivity in the dystrophic retinas was significantly decreased by the prolonged denervation of the neuroretina (**g**).

h,i. Representative images of protein kinase Cα (PKCα)-positive rod BCs in the INL and IPL in age-matched WT and rd10 mice (**h**). The deviation of rod BC axons from the physiological right angle to the GCL (**i**, *left panel*) and the mean and standard deviation of the IPL width (**i**, *right panels*) were quantified. In old rd10 mice, the rod BC axons were markedly deviated from the right angle, and the IPL was thinner and less organized than in the age-matched WT controls.

Scale bars, 50 µm. Data are shown as means $\pm$ sem (n = 5 for both WT and rd10 mice). *p<0.05, **p<0.01, ***p<0.001; unpaired Student's *t*-test.

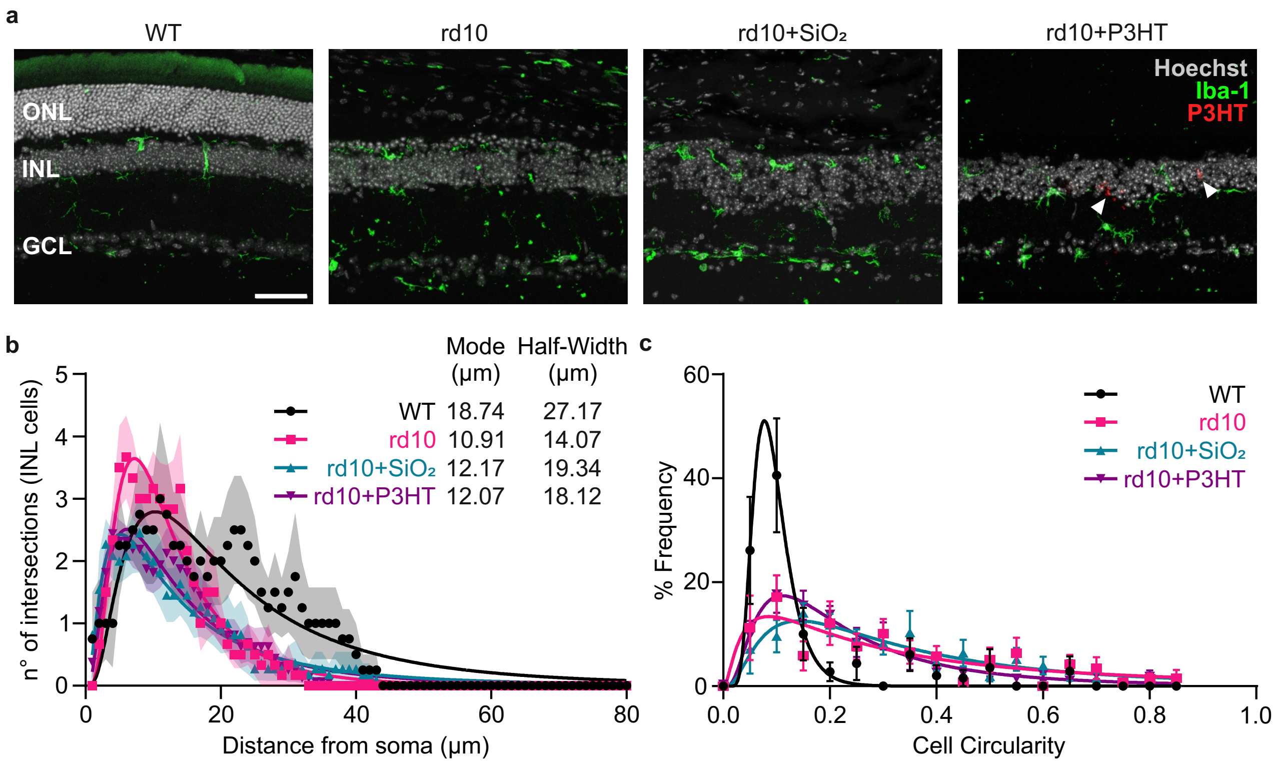

Figure S4. Injection of P3HT-NPs in old rd10 mice does not affect the activation state of Iba-1-positive retinal microglial cells.

a. Representative transverse sections of retinas dissected from WT mice and homozygous rd10 mice that were non-injected (rd110), sham-injected with SiO_2-NPs (rd10+SiO_2), or injected with P3HT-NPs (rd10+P3HT). Sections were subjected to Iba-1 staining (green) to identify microglial cells and nuclear staining with Hoechst 33258 (white). Injected P3HT-NPs (arrowheads) are detected by their autofluorescence (purple). Scale bar, 50 µm.

b. The extent of ramification of Iba-1-positive microglial cells in the INL was evaluated by Sholl analysis. The minor diameter of the cell soma was used as the starting radius, the soma was removed from the segmented image, and intersections were counted at 1-µm increasing distances from the soma using concentric circles. The resulting intersection profile is plotted as a function of distance from the soma and fitted with a log-normal curve. The maximal branching span was determined by calculating the mode and half-width of the four curves. A consistent decrease in the extent of microglial ramification was observed in all dystrophic rd10 groups, irrespective of the treatments. The analysis was performed on n = 4, 6, 11, and 16 microglial cells from n = 6, 6, 8, and 9 mice, respectively, for the WT, rd10, rd10+SiO_2, and rd10+P3HT groups.

c. A shape descriptor correlating to the activation state of Iba-1-positive microglial cells is the circularity index, ranging from zero (elongated polygon) to 1 (perfect circle). The averaged frequency distribution histograms (means ± sem) were fitted with a log-normal curve. The similar increase in the circularity index observed in all dystrophic rd10 groups, regardless of the treatments, compared to the WT group, indicates a decrease in ramification and a tendency to assume an ameboid-like shape in the presence of the degenerative process. n = 7, 7, 9, and 9 mice, respectively, for the WT, rd10, rd10+SiO_2, and rd10+P3HT groups.

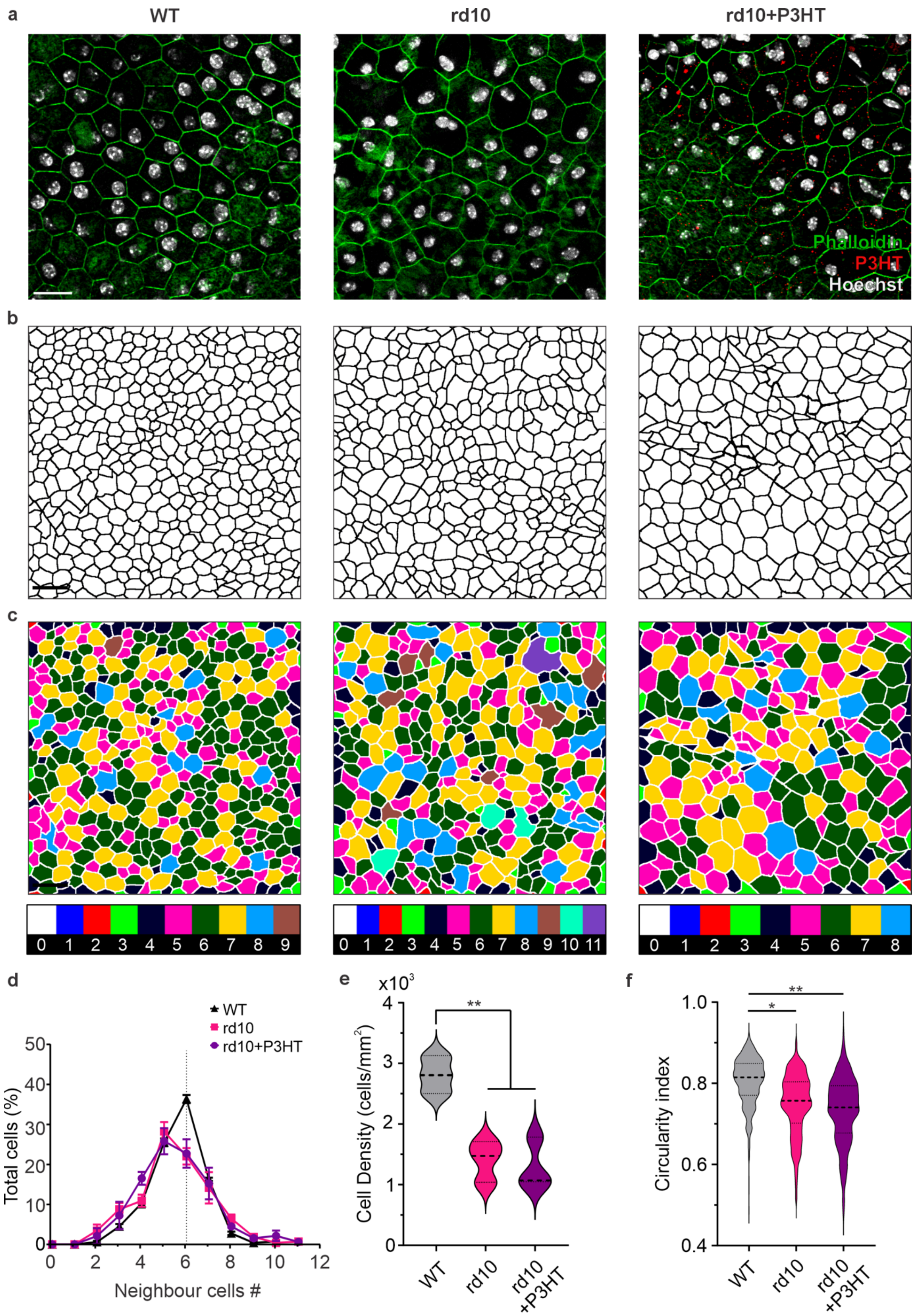

Figure S5. Injection of P3HT-NPs in old rd10 mice does not affect the morphology of RPE cells.

a. Representative images of the RPE monolayer in whole-mount retinas of aged-matched control mice (WT), untreated dystrophic mice (rd10), and P3HT-NP-injected rd10 mice (rd10+P3HT),

stained with Texas Red-Phalloidin (green) and Hoechst 33258 (white). P3HT-NPs are in red. Scale bar, 25 μm.
b. Representative maps of WT, rd10, and rd10+P3HT RPE flat mounts with cell borders segmented for each RPE cell. Scale bar, 50 μm.
c. Classification of RPE cells based on the number of neighboring cells. Each color represents the number of adjacent cells contacted by the single RPE cell. Color codes are shown below. Scale bar, 50 μm.
d. Frequency of RPE cells (in percent of the total number of analyzed cells) as a function of the number of contacted neighboring cells. WT animals display a higher number (peak = 6) of neighboring cells with respect to rd10 mice, regardless of the treatment.
e. The RPE cell density (cells/mm^2) is expressed as the number of cells per square millimeter. Both rd10 and rd10+P3HT groups have a significantly lower cell density compared to WT mice. $^{**}p<0.01$; one-way ANOVA/Tukey's multiple comparison tests (n=3 for each group).
f. The plot of RPE cell circularity values reveals higher values in WT animals with respect to rd10 mice, regardless of the treatment. A perfect hexagon would have an index of 0.907. $^*p<0.05$, $^{**}p<0.01$; one-way ANOVA/Fisher's LSD multiple comparison tests (n=3 for each group).

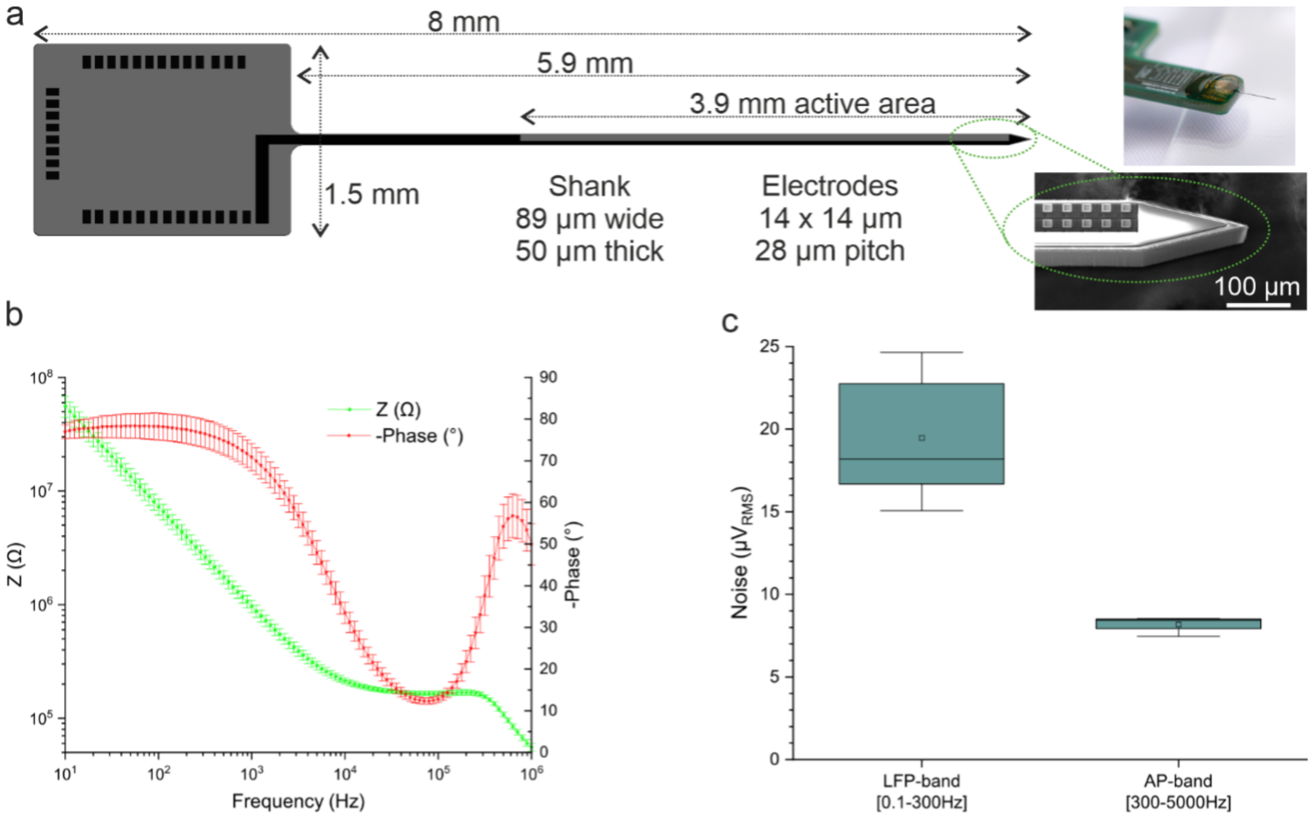

Figure S6. Characterization of SiNAPS 256-channel neural probe.
a. Schematic representation of a SiNAPS 256-channel neural probe indicating the main dimensions with a probe tip zoom image acquired by scanning electron microscopy (SEM). A view of the mounted probe used in the experiments is shown in the top-right corner.
b. Electrochemical Impedance Spectroscopy (EIS) of the electrodeposited platinum electrodes. The 256 electrodes were short-circuited for the measurement, and the impedance module value was estimated by multiplying the result by the number of electrodes (256). The mean electrode impedance module at 1 kHz was 967 ± 117 kΩ/electrode (n = 5 probes × 256 electrodes, mean ± SD).
c. Box plot (dot, mean; line, median; box, 25-75%; whiskers, range within 1.5 IQR) quantifying the root mean-square (RMS) noise measured in saline. The measured values are 19.46 ± 4.07 μV_{RMS} (LFP-band) and 8.17 ± 0.47 μV_{RMS} (AP-band) (n = 5 probes × 256 electrodes, mean ± SD).

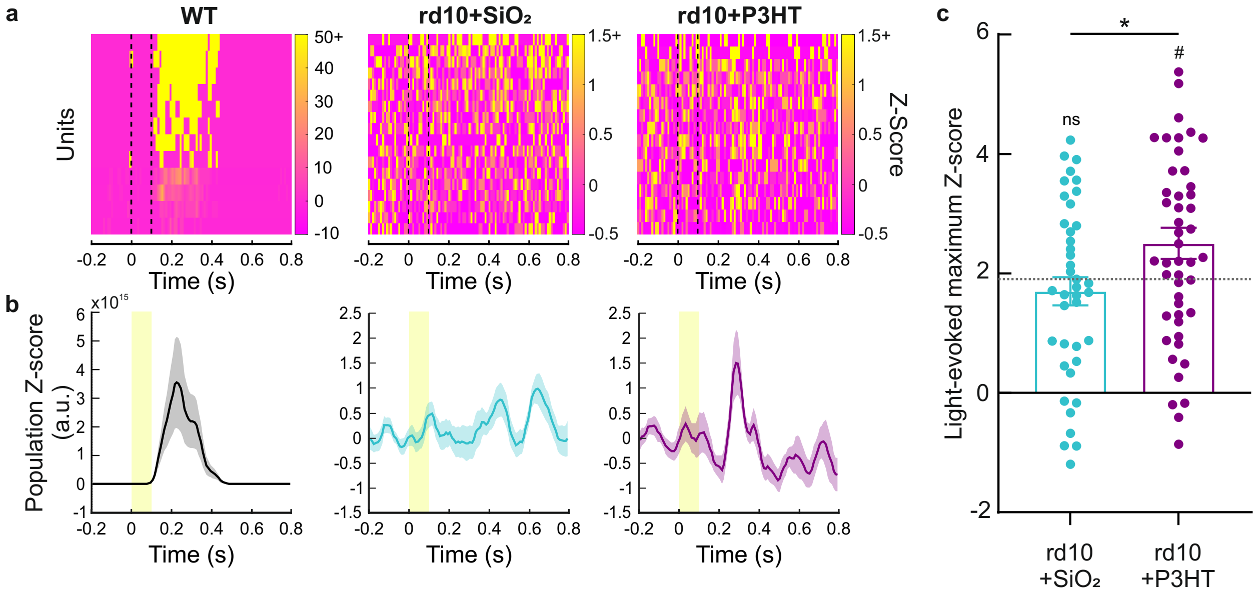

Figure S7. Retina reactivation by P3HT-NPs recovers light-evoked firing activity in old rd10 mice.

a. Z-score heatmap for all sorted neuronal units in the peri-stimulus time window (black dashed lines indicate stimulus) for WT and rd10 mice injected with either SiO_2-NPs (rd10+SiO_2) or P3HT-NPs (rd10+P3HT). For further details, see legend to Figure 7 (bin = 10 ms).

b. Average Z-score trends in the same peri-stimulus time window (yellow boxes indicate flashes, shaded areas indicate ± sem, bin = 10 ms, smoothing window = 50 ms).

c. Quantification of the maximum firing rate activity Z-score in the post-stimulus time window (0.15-0.5 s). Colored bars indicate the means ± sem of the rd10+SiO_2 and rd10+P3HT groups, while the dashed grey line indicates 1.96, the statistical significance threshold for z-scores (ns, p=0.089 for rd10+SiO_2; #, p=0.012 for rd10+P3HT; one-sample Student's *t*-test *versus* the z-score threshold). *p=0.0279, unpaired Student's *t*-test rd10+P3HT versus rd10+SiO_2 (n = 40,48 neuronal units from n = 5 and 6 animals for rd10+SiO_2 and rd10+P3HT groups, respectively).
